# Can the Quality of Semen Affect the Fertilisation Indices of Turkey Eggs?

**DOI:** 10.3390/ijms262211000

**Published:** 2025-11-13

**Authors:** Aleksandra Orzołek, Anna Dziekońska, Paulina Skorynko, Joanna Ner-Kluza

**Affiliations:** 1 Department of Animal Biochemistry and Biotechnology, Faculty of Animal Bioengineering, University of Warmia and Mazury in Olsztyn, Oczapowskiego 5, 10-719 Olsztyn, Poland; a.dziekonska@uwm.edu.pl (A.D.); paulinaskorynko28@wp.pl (P.S.); 2 Department of Analytical Chemistry and Biochemistry, Faculty of Materials Science and Ceramics, AGH University, Mickiewicza 30, 30-059 Kraków, Poland; nerjoanna@gmail.com

**Keywords:** turkey, fertility, antioxidants, oxidative stress, proteome composition

## Abstract

Several factors, including semen quality, can influence fertilisation success. Poor semen parameters may necessitate more frequent inseminations or the removal of males with consistently low fertility. This study evaluated turkey ejaculates (*n* = 37) with good fertility (GF) and impaired fertility (IF). The analyses included sperm motility parameters (total motility—TMOT, progressive motility—PMOT, curvilinear velocity—VCL, straight-line velocity—VSL, average path velocity—VAP, linearity—LIN, straightness—STR, amplitude of lateral head displacement—ALH, and beat cross frequency—BCF), plasma membrane integrity (PMI), mitochondrial membrane potential (MMP), and nitric oxide (NO) production, as well as enzymatic and biochemical assays of semen, such as superoxide dismutase (SOD), glutathione peroxidase (GPx), catalase (CAT) activities, glutathione (GSH) content, malondialdehyde (MDA) levels, and zinc (Zn^2+^) concentration. In parallel, the proteomes of seminal plasma and spermatozoa were separated using SDS- and Tricine-PAGE, and selected proteins were identified by nano LC-MS/MS. Spermatozoa derived from IF ejaculates exhibited significantly reduced TMOT (*p* = 0.002), VCL (*p* = 0.028), and PMI (*p* = 0.000), accompanied by elevated STR (*p* = 0.000) and NO production (*p* = 0.044). In the seminal plasma of IF males, a significant decrease was noted in SOD (*p* = 0.000) and GPx (*p* = 0.001) activities, whereas CAT activity was markedly higher (*p* = 0.014). Seminal fluid from IF ejaculates was also characterised by increased GSH (*p* = 0.014) and MDA (*p* = 0.014) concentrations, accompanied by reduced Zn^2+^ content (*p* = 0.014). In contrast, IF spermatozoa exhibited elevated SOD activity (*p* = 0.001), but reduced GPx (*p* = 0.000) and CAT (*p* = 0.012) activities. Sperm cells from IF ejaculates also had lower GSH levels (*p* = 0.000), higher MDA concentrations (*p* = 0.000), and increased Zn^2+^ content (*p* = 0.018) compared with those from GF ejaculates. A proteomic analysis revealed differences in fertility-associated proteins: peroxiredoxin 6 (PRDX6) was detected exclusively in GF semen, whereas alpha-enolase (ENO1), fatty acid-binding protein (FABP7), cytoplasmic aspartate aminotransferase (GOT1), and L-lactate dehydrogenase B (LDHB) were detected only in IF semen. Overall, the results demonstrate that both semen parameters and proteome composition may potentially affect the fertilisation outcomes in turkeys.

## 1. Introduction

Reproduction in turkeys (*Meleagris gallopavo*) is affected by factors related to egg fertility and hatchability. These two main parameters are influenced by genetic and environmental factors [1]. During production, farms aim to maximise the number of fertilised hatching eggs. A decrease below the acceptable threshold of fertilised eggs results in significant economic losses and necessitates investigation into the cause.

The evaluation of turkey semen quality extends beyond a visual assessment of the ejaculate and includes the analysis of various biochemical parameters. These biochemical indicators are associated with multiple functions of the male reproductive tract and are considered more sensitive measures of semen quality than conventional quantitative parameters [2]. In turkeys, semen of reduced quality is typically characterised by an increased proportion of abnormal spermatozoa, spermatids, and spermiophages [3], accompanied by elevated activities of aspartate aminotransferase, acid phosphatase (AcP), and superoxide dismutase (SOD), as well as an increased total protein concentration [4,5]. The biochemical profile of seminal plasma can also include measurements of cholesterol concentration and the activities of AcP, aspartate aminotransferase, amidase, and antiproteinase (AaP). Alterations in these parameters have been linked to a decline in semen quality and the occurrence of yellow semen syndrome (YSS) [2]. The elevated AcP activity observed in turkey semen may be associated with damaged spermatozoa [6] or AcP secretion by ductal epithelial cells [7,8]. Proteomic analyses have revealed distinguishing characteristics between white (normal) and yellow (low-quality) seminal plasma. Słowińska et al. [9] reported that the expression levels of transthyretin, serum albumin-like protein, hemopexin-like protein, and immunoglobulin light chain V-J-C were approximately three times higher in YSS-affected samples compared with normal seminal plasma. More recent findings by Rafalska et al. [10] demonstrated that yellow seminal plasma exhibits significantly higher total protein content, glutathione (GSH) and malondialdehyde (MDA) concentrations, as well as increased activities of alkaline phosphatase (ALP), AcP, and glutathione peroxidase (GPx). Furthermore, the cited authors found that the phosphorylation profiles of both seminal plasma and spermatozoa proteins vary depending on ejaculate quality (white vs. yellow) [10,11]. The proteins exhibiting differential phosphorylation included those encoded by the following genes: *SPARC* (SPARC protein), *PPIB* (peptidyl-prolyl cis/trans isomerase B), *TRFE* (ovotransferrin), *QSOX1* (sulfhydryl oxidase 1), *PRDX1* (peroxiredoxin 1), *PRDX6* (peroxiredoxin 6), *FASN* (fatty acid synthase), *CKB* (creatine kinase subtype B), *ORM2* (acid glycoprotein α1), *APOA1* (apolipoprotein A1), *SSC5D* (SSC5D-1-like soluble scavenger receptor cysteine-rich domain containing protein SSC5D), *RAP1B* (Ras-related protein Rap-1b), *CDC42* (cell division control protein 42 homolog), *FTH* (ferritin heavy chain), *TBB* (tubulins), *CKMT2* (creatine kinase S-type), *VDAC2* (voltage-dependent anion-selective channel protein 2), *UBB* (polyubiquitin-B), *GAPDH* (glyceraldehyde-3-phosphate dehydrogenase), *TTR* (transthyretin), *RBP4* (retinol-binding protein 4), and *TPI1* (triosephosphate isomerase). In addition, Pardyak et al. [12] identified alterations in four proteins implicated in fertilisation and indirectly associated with sperm quality, i.e., acrosin (ACR), 1-phosphatidylinositol 4,5-bisphosphate phosphodiesterase zeta (PLCZ1), EF-hand domain-containing protein 2 (EFHC2), and Ca^2+^-binding tyrosine-phosphorylation regulated protein (CABYR). Despite these advances, the proteomic landscape of turkey semen remains insufficiently characterised.

The existing body of research is limited and primarily aims to elucidate the molecular determinants underlying reduced ejaculate quality and, consequently, diminished suitability for artificial insemination (AI). The importance of semen evaluation for selecting males or monitoring reproductive performance is well-recognised in poultry breeding [8]. Lower values of fertilisation indices in turkey eggs may result from inferior ejaculate quality, which is usually associated with reduced levels of biological and biochemical parameters of semen, often with a specific proteome composition. This study aimed to analyse differences in the biological, biochemical, and protein profiles of ejaculates from turkeys with good fertility (GF) and impaired fertility (IF).

## 2. Results

### 2.1. Parameters of Sperm Functionality

Statistically significant and highly significant differences were noted in selected quality parameters of spermatozoa from GF and IF turkey ejaculates (Table 1). Sperm concentration was higher, but total motility (TMOT) was lower in IF ejaculates. However, no differences in the progressive motility (PMOT) of spermatozoa were observed. The straight-line rectilinear velocity (VSL) and straightness (STR) were greater in IF ejaculates, whereas curvilinear velocity (VCL) was higher in GF ejaculates. Other sperm motility indices, including the average path velocity (VAP), the amplitude of lateral head displacement (ALH), and beat-cross frequency (BCF), did not differ significantly between the analysed groups. The IF group was characterised by lower sperm plasma membrane integrity (PMI) and a higher percentage of nitric oxide (NO)-generating sperm. No significant differences in sperm mitochondrial membrane potential (MMP) were observed between the examined groups (Table 1).

### 2.2. Antioxidant Status of Turkey Semen

The study revealed significant differences in the antioxidant status of seminal plasma from GF and IF ejaculates. In the seminal plasma of GF ejaculates, SOD and GPx activities were higher, whereas catalase (CAT) activity was lower. In addition, GSH levels and MDA content were reduced in seminal plasma from GF ejaculates. Furthermore, zinc (Zn^2+^) levels were higher in seminal plasma from GF ejaculates compared to IF ejaculates (Table 2).

Superoxide dismutase activity was enhanced, whereas GPx activity was reduced in spermatozoa from IF ejaculates. In turn, higher CAT activity was observed in GF ejaculates. Notably, spermatozoa from IF ejaculates exhibited lower GSH levels and higher MDA levels. Zinc content showed the opposite trend and was higher in spermatozoa from IF ejaculates (Table 3).

### 2.3. Differences in the Proteomic Profiles of Seminal Plasma and Spermatozoa from Ejaculates with Good Fertility and Impaired Fertility

**Figure 1:**
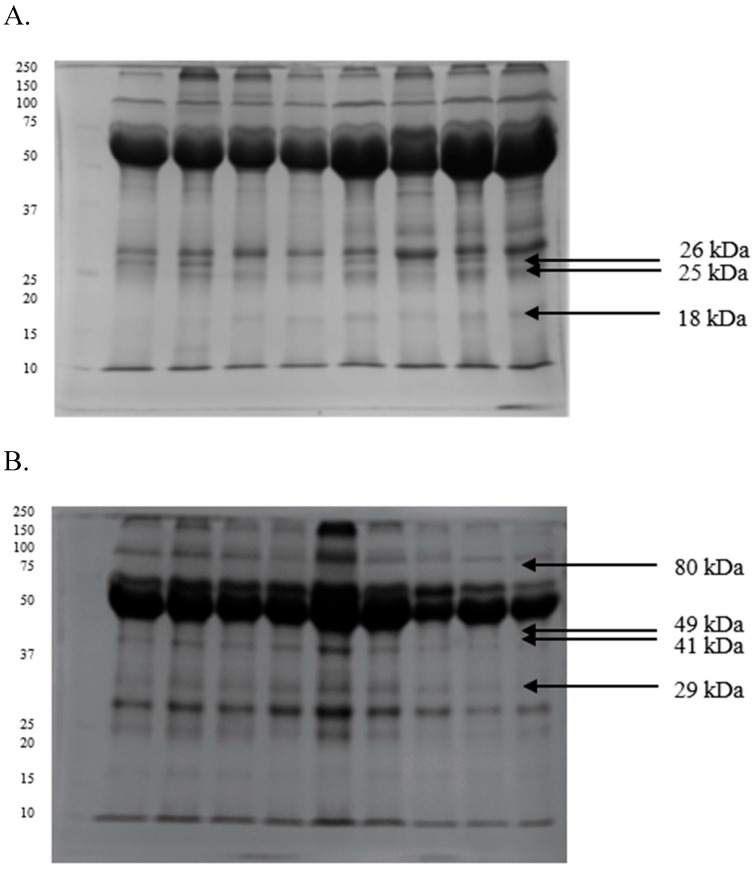
Proteomes of seminal plasma derived from turkey ejaculates with good (**A**) and impaired (**B**) fertility, separated by SDS-PAGE electrophoresis.

**Figure 2:**
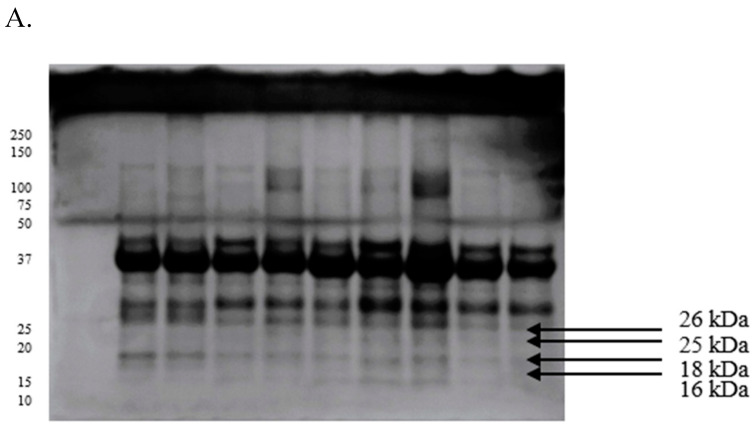

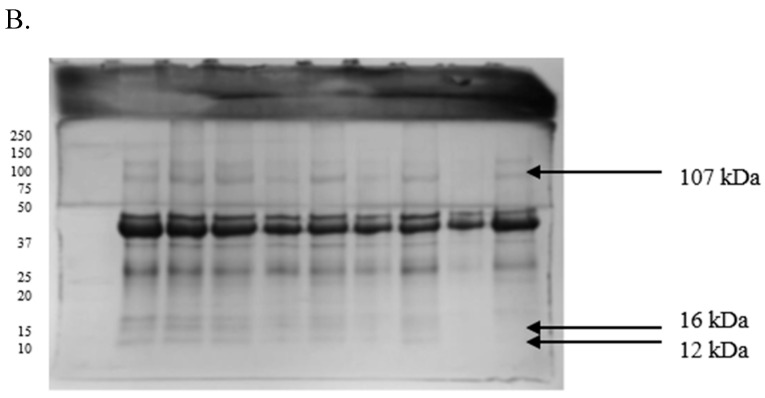
Proteomes of seminal plasma derived from turkey ejaculates with good (**A**) and impaired (**B**) fertility, separated by Tricine-PAGE electrophoresis.

In the SDS-PAGE analysis, approximately 25 proteins, including the three most abundant ones with prominent bands at 26, 25, and 18 kDa, were identified in the seminal plasma of GF semen (Figure 1A). In turn, polypeptides with prominent bands at 80, 49, 41, and 29 kDa were detected in the seminal plasma of IF ejaculates (Figure 1B). A three-layer gel with the addition of tricine supported the separation of protein fractions with a molecular weight below 70 kDa. Four polypeptide bands at around 26, 25, 18, and 16 kDa were observed in the seminal plasma from GF sperm (Figure 2A). Two prominent bands at 107 and 12 kDa were additionally identified in the seminal plasma of IF ejaculates (Figure 2B).

**Figure 3:**
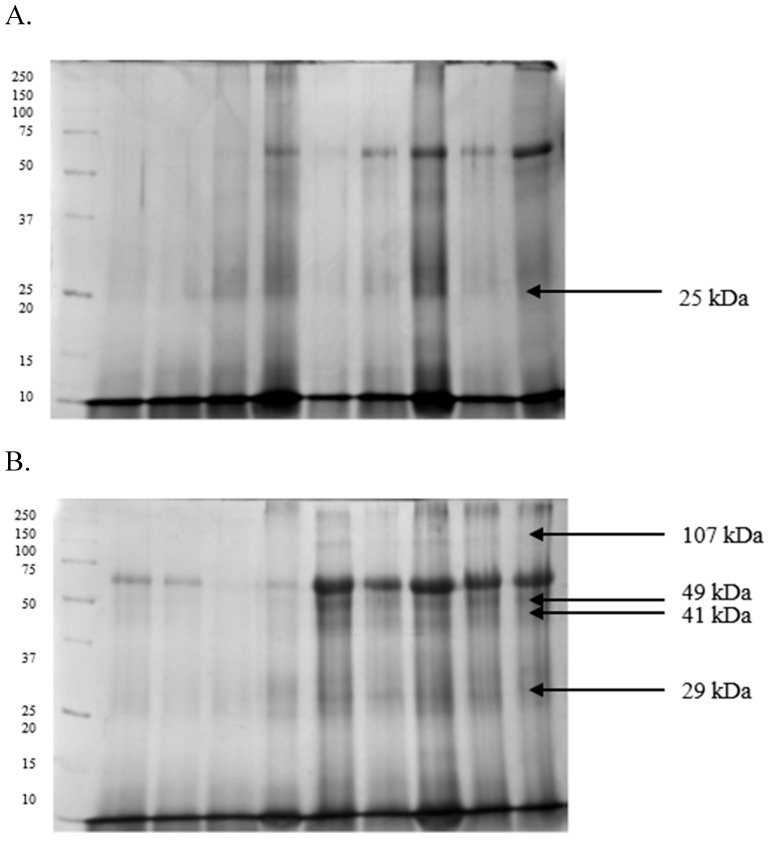
Proteomes of spermatozoa derived from turkey ejaculates with good (**A**) and impaired (**B**) fertility, separated by SDS-PAGE electrophoresis.

**Figure 4:**
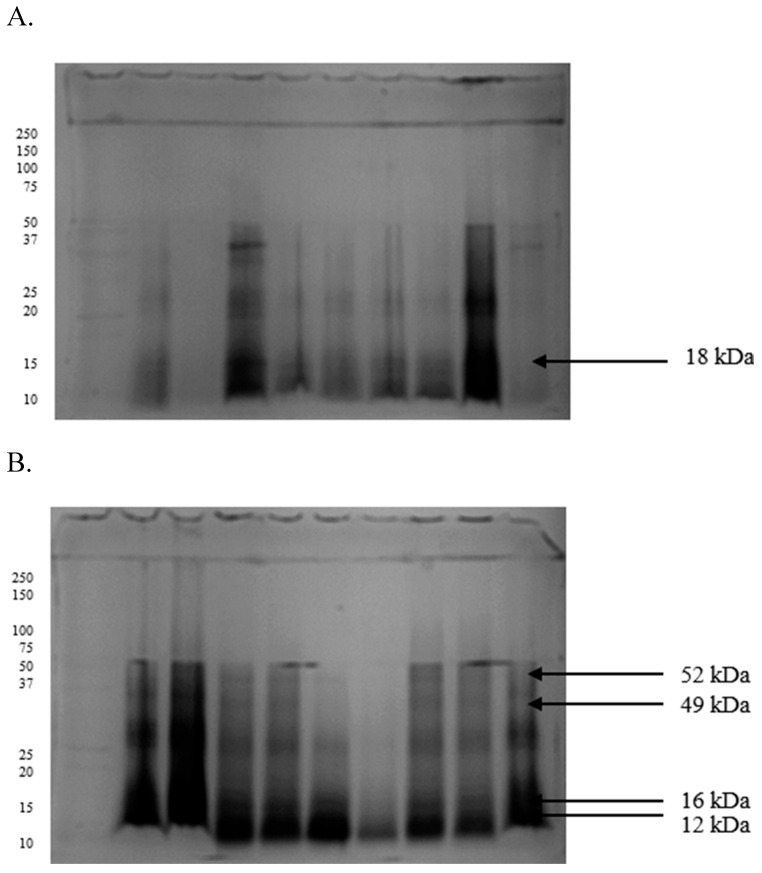
Proteomes of spermatozoa derived from turkey ejaculates with good (**A**) and impaired (**B**) fertility, separated by Tricine-PAGE electrophoresis.

In spermatozoa, the separations were less accurate, and the isolated fractions were less defined. The amount of protein separated in the gels was significantly lower, producing poorer profiles with a maximum of 15 bands (Figure 3 and Figure 4). However, a 25 kDa polypeptide was identified in spermatozoa from GF turkey ejaculates (Figure 3A). At the same time, 107, 49, 41, and 29 kDa proteins were detected in IF ejaculates (Figure 3B). The Tricine-PAGE analysis revealed an 18 kDa protein in high-quality spermatozoa (Figure 4A) and four prominent protein bands at 52, 49, 16, and 12 kDa in low-quality semen (Figure 4B).

### 2.4. Protein Identification Results

Selected bands were excised based on differences in their optical density (OD) (Table 4 and Table 5).

A comprehensive list of the proteins identified in GF and IF semen is provided in the Supplementary Materials (Tables S1–S4). A total of 62 proteins were detected in seminal plasma associated with good fertility. Among these, *ACT5*, *ACTB*, *ACTG1*, *ACTG2*, *AK1*, *CA2*, *CKMT2*, *CFAP20*, *FTH*, *HAGH*, *HPRT1*, *HSP70*, *PCMT1*, *PGAM1*, *PPIB*, *PRDX6*, *PSMB5*, *RAB10*, *RAB5B*, *RAB5C*, *RBP4*, *SDHB*, *SPATA18*, *TBA4*, *TBB1*, *TBB2*, *TBB3*, *TBB6*, *TBB7*, *TPI1*, *UQCRFS1*, *YWHAB*, *YWHAE*, *YWHAQ*, and *ZPBP1* were specifically identified in seminal plasma derived from GF ejaculates. In turn, a total of 65 proteins were identified in the seminal plasma of individuals with impaired fertility. The proteins encoded by the following genes: *ACO1*, *ACTN4*, *ANPEP*, *ANXA2*, *ANXA5*, *ATP5F1B*, *B2M*, *BLMH*, *CYC*, *CYT*, *EEF1A*, *EEF2*, *EIF4A2*, *ENO1*, *ENO2*, *FABP7*, *FGB*, *GLG1*, *GOT1*, *HSP90B1*, *LAC*, *LAMB1*, *LAMP1*, *LDHA*, *LDHB*, *MDH1*, *NDK*, *NEGR1*, *NEL*, *NHERF1*, *PAFAH1B1*, *PSAP*, *PTPRG*, *RAB2A*, *SEC22B*, *SERPINI1*, *TBA5*, and *TGFB2* were identified in IF seminal plasma. The proteome shared by seminal plasma from the GF and IF groups consisted of polypeptides encoded by the following genes: *ACR*, *ALB*, *APOA1*, *ASTL*, *CCT8*, *CKB*, *CLU*, *COL12A1*, *GAPDH*, *GSN*, *HSP90AA1*, *HSPA8*, *LYZ*, *PLCZ1*, *QSOX1*, *RPS27A*, *SPARC*, *TBB4*, *TBB5*, *TF*, *TTR*, *TSN*, *TUBA1*, *UBB*, *VDAC2*, *YWHAG*, and *YWHAZ*.

In turn, a total of 46 proteins were identified in GF spermatozoa. Some of these proteins were observed only in this type of sperm cells, including ACTG1, AK1, CA2, GST5, HSP70, HSP90AA1, NHERF1, PKM, PRDX6, QSOX1, RAB10, RAB5B, RAB5C, TBA5, TBB1, TPI1, TUBA1, UQCRFS1, and YWHAZ. In contrast, spermatozoa from IF ejaculates contained a total of 49 proteins, including ACAA2, ACADM, ACAD2, ACTB, ACTG2, ANPEP, ATG4B, ATP13A4, CALM, CFAP20, EEHC2, ENO1, FABP7, FN1, GOT1, GOT2, GSN, HSPD1, LAC, LDHB, NEL, PGK, PPP1CB, SDHB, SPAG6, and SPAG16. The proteome shared by spermatozoa from the GF and IF ejaculates included polypeptides encoded by the following genes: *ACR*, *ATP5B*, *ALB*, *APOA1*, *ASTL*, *ATP5B*, *CKB*, *CKMT2*, *CLU*, *COL12A1*, *GAPDH*, *HSPA8*, *ODF2*, *PGAM1*, *RPS27A*, *SPATA18*, *TBA2*, *TBA4*, *TBB2*, *TBB3*, *TBB4*, *TBB5*, *TBB7*, *TF*, *UBB*, and *VDAC2*.

**Figure 5:**
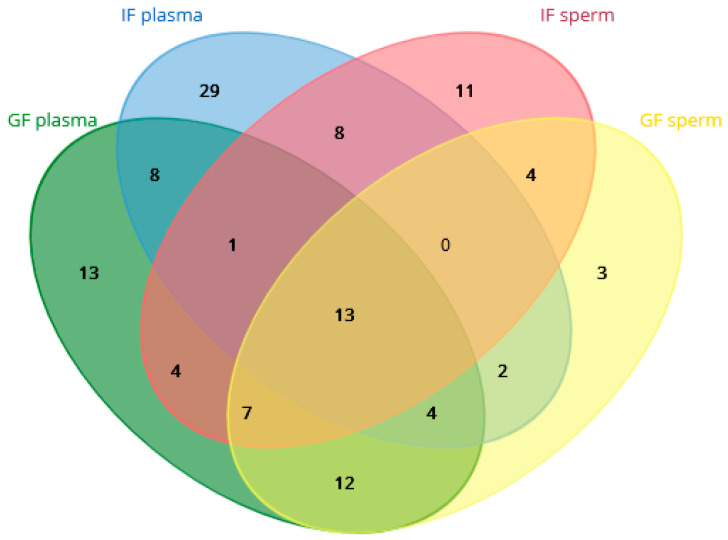
Diagram presenting the number of proteins shared between seminal plasma and spermatozoa from turkey ejaculates with good fertility (GF) and impaired fertility (IF).

The proteome (the complete set of individual proteins) shared between different components of GF and IF ejaculates is shown in Figure 5. Seminal plasma and spermatozoa from the GF group shared proteins encoded by ACR, ACT5, ACTG1, AK1, ALB, APOA1, ASTL, CA2, CKB, CKMT2, CLU, COL12A1, GAPDH, HAGH, HPRT1, HSP70, HSPA8, HSP90AA1, PCMT1, PGAM1, PRDX6, QSOX1, RAB10, RAB5B, RAB5C, RPS27A, SPATA18, TSN, TBA2, TBA4, TBB1, TBB2, TBB3, TBB4, TBB5, TBB6, TBB7, TF, TPI1, TUBA1, UBB, UQCRFS1, VDAC2, and YWHAZ. In turn, seminal plasma and spermatozoa from the IF group shared proteins encoded by ACR, ALB, ANPEP, APOA1, ASTL, CKB, CLU, COL12A1, ENO1, FABP7, GAPDH, GSN, GOT1, HSPA8, LAC, LDHB, NEL, RPS27A, TBB4, TBB5, TF, UBB, and VDAC2. Interestingly, the proteins identified in seminal plasma from GF ejaculates were present in IF sperm, whereas the proteins detected in IF plasma were found in GF sperm. Numerous proteins were shared between GF plasma and IF sperm, including ACR, ACT5, ACTB, ACTG2, ALB, APOA1, ASTL, CKB, CKMT2, CFAP20, CLU, COL12A1, GAPDH, GSN, HSPA8, HSP70, PGAM1, RPS27A, SDHB, SPATA18, TBA4, TBB2, TBB3, TBB4, TBB5, TBB7, TF, UBB, and VDAC2. In turn, the proteins shared between IF plasma and GF sperm included ACR, ALB, APOA1, ASTL, CKB, CLU, COL12A1, GAPDH, HSP90AA1, HSPA8, NHERF1, QSOX1, RPS27A, TBA5, TBB4, TBB5, TF, TUBA1, UBB, VDAC2, and YWHAZ. Therefore, the following proteins were shared between GF plasma, IF plasma, GF sperm, and IF sperm: ACR, ALB, APOA1, ASTL, CKB, CLU, COL12A1, GAPDH, HSPA8, RPS27A, TBB4, TBB5, TF, UBB, and VDAC2.

Some proteins were identified only in specific components of semen from the examined quality groups. For instance, FTH and HAGH were detected in several bands in seminal plasma from the GF group, whereas B2M was present in seminal plasma from the IF group. No proteins were consistently detected in GF sperm, whereas EFHC2 and GOT2 were identified in IF sperm. Furthermore, some proteins were identified in seminal plasma and spermatozoa from one group only. For example, PRDX6 was detected only in GF semen, whereas ENO1, FABP7, GOT1, and LDHB were present only in IF semen.

### 2.5. Gene Ontology Results

**Figure 6:**
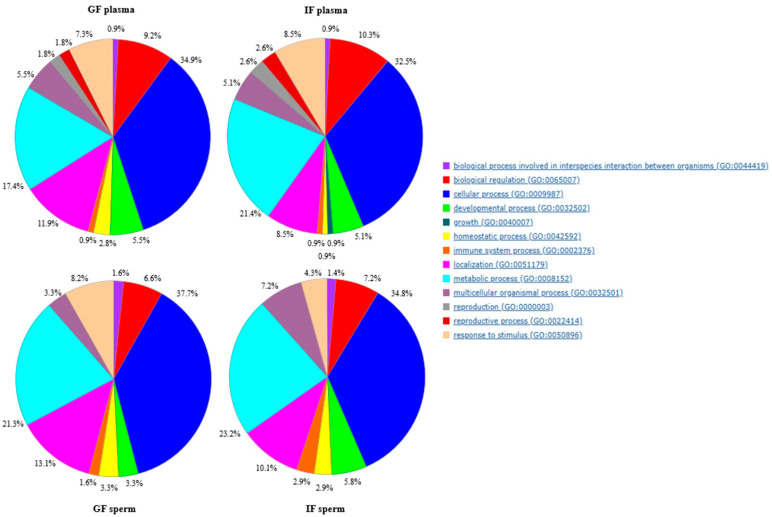
Classification and comparison of semen proteins from turkey ejaculates with good fertility (GF) and impaired fertility (IF) according to the Gene Ontology term Biological Process.

**Figure 7:**
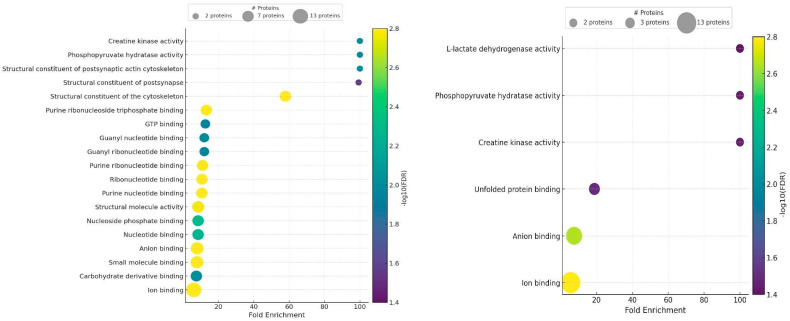
Main Molecular Functions attributed to seminal plasma proteins in good fertility (GF) (**left**) and impaired fertility (IF) (**right**) ejaculates.

The Gene Ontology (GO) analysis revealed that the proteomes of seminal plasma and spermatozoa were very similar (Figure 6) and differed mainly in the content of individual protein fractions responsible for specific GO processes. Seminal plasma from both quality groups differed in the presence or absence of a protein associated with growth (GO: 0040007), which was detected in seminal plasma from IF ejaculates. The GO analysis revealed that seminal plasma was predominantly composed of proteins associated with cellular and multicellular processes, metabolism, localisation, immunity, reproduction, and regulation (Figure 6). The Molecular Function analysis of the proteins identified in GF seminal plasma demonstrated that most of them were involved in creatine kinase and phosphopyruvate hydratase activity and served as structural components of selected biomolecules. These proteins were also involved in binding various substances, in particular nucleotides and ions (Figure 7). The proteins present in IF seminal plasma were generally involved in creatine kinase and phosphopyruvate hydratase activity, L-lactate dehydrogenase activity, and binding of unfolded proteins and anions (Figure 7).

**Figure 8:**
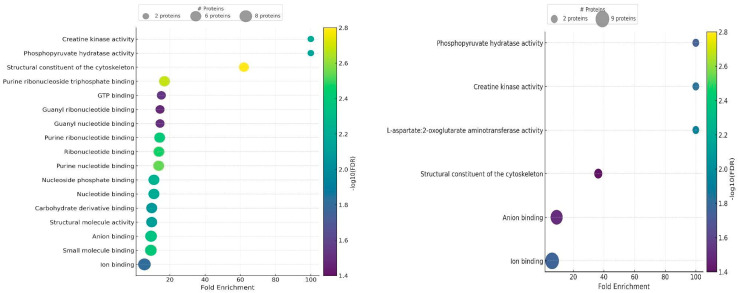
Main Molecular Functions attributed to spermatozoa proteins in good fertility (GF) (**left**) and impaired fertility (IF) (**right**) ejaculates.

The proteins identified in the GO analysis of sperm proteomes were mainly involved in the same biological processes as seminal plasma proteins, but none were associated with reproductive functions. The analysis also showed a higher percentage of polypeptides involved in localisation and metabolic processes (Figure 6). In the Molecular Function analysis, some of these proteins had anion and ion binding capabilities. The proteins detected in GF sperm were involved in the same processes as those identified in GF seminal plasma (Figure 8). In contrast, most proteins identified in IF sperm were unique to this quality group. In IF ejaculates, the proteins detected in seminal plasma and spermatozoa differed mainly in enzymatic activity (dehydrogenases in plasma and aminotransferases in sperm) and other protein functions with a high false discovery rate (FDR), such as the binding of unfolded proteins in seminal plasma and serving as structural components of the cytoskeleton in spermatozoa (Figure 8).

A complete list of proteins identified in seminal plasma and spermatozoa from GF and IF ejaculates and involved in various biological processes (GO) is provided in the Supplementary Materials (Tables S5 and S6).

## 3. Discussion

The main goal of turkey breeding is to increase the production of high-quality hatching eggs, thereby raising the number of poults obtained. Fertility is measured as the percentage of eggs with normally developing embryos, while eggs showing no signs of development are classified as “clear eggs” [13]. Accurate data support comparisons of hatchery performance and help identify fertility issues by revealing abnormal patterns.

### 3.1. Potential Impact of Oxidative Stress on IF Semen

High accumulation of reactive oxygen species (ROS) in semen impacts sperm functionality and, consequently, the fertilising potential of the ejaculate. Increased ROS production and insufficient antioxidant defence mechanisms lead to a redox imbalance and intracellular oxidative stress. This process involves lipid peroxidation, loss of plasma membrane integrity, greater membrane permeability, reduced sperm motility, and structural damage to sperm DNA, which ultimately leads to sperm apoptosis [14,15]. Spermatozoa are particularly vulnerable to the harmful effects of ROS because their cell membranes contain high levels of unsaturated fatty acids. Thus, the balance between ROS generation and ROS scavenging affects sperm viability and the fertilising capacity of spermatozoa [16].

Sperm viability can be assessed using various microscopic and cytometric techniques. Common methods include vital staining assays, such as the eosin–nigrosin test and the hypo-osmotic swelling test (HOST) [17]. Additionally, fluorescent dye-based assays, such as Hoechst, carboxyfluorescein diacetate (CFDA), or SYBR-14, may be employed [18]. For instance, using SYBR-14 combined with propidium iodide (PI) enables more accurate differentiation between live and dead sperm cells. This method is based on the principle that intact sperm membranes prevent certain dyes from entering the cell, whereas damaged membranes allow dye penetration and staining [19]. All of the aforementioned methods provide valuable insights into sperm plasma membrane integrity, a key indicator of cell functionality. The integrity of the plasma membrane is fundamental to sperm fertility, as it underpins essential physiological processes including capacitation, the acrosome reaction, and binding to the zona pellucida [20]. In poultry sperm, the most common dual-staining approaches are eosin-nigrosin and SYBR-14 combined with PI [21]. In the present study, sperm viability parameters were compromised in the ejaculates of IF individuals. These ejaculates were characterised not only by lower TMOT and VCL, and a higher percentage of NO-producing sperm, but also by significantly lower PMI. The above could point to a decline in gamete function and, possibly, intensified oxidative processes in semen. In poultry, sperm motility decreases in response to semen oxidation [22]. Higher sperm concentration in IF turkey ejaculates may have reduced motility by causing an excessive accumulation of gametes per unit volume, impairing cell viability, and increasing oxidative stress in semen. Superoxide dismutase, CAT, and GPx constitute the primary antioxidant system in semen. These metalloenzymes are found in both intracellular and extracellular spaces [23]. Statistically significantly higher SOD and GPx activities were observed in the seminal plasma and spermatozoa of GF ejaculates, which may suggest that spermatozoa received additional antioxidant protection from the external environment. At the same time, CAT was prevalent in IF ejaculates. Research has confirmed that SOD provides protection against heat stress, refrigeration, exposure to toxic agents, and other conditions associated with oxidative stress in poultry production [24]. Thus, higher SOD activity in the seminal plasma of fertile turkeys may indicate better antioxidant protection within the system. It can be assumed that higher Zn^2+^ levels in seminal plasma from GF ejaculates enhanced SOD activity, as this trace element is a cofactor of copper/zinc superoxide dismutase (Cu/Zn SOD) [25]. Thus, low Zn^2+^ levels in the seminal plasma of IF turkeys could decrease extracellular Cu/Zn-specific SOD activity, thereby increasing ROS generation. The present work suggests that GPx activity can be influenced by the action of other enzymes. Higher CAT activity in IF ejaculates could suggest that this enzyme compensates for a deficiency in GPx activity. Crisol et al. [26] proposed that seminal plasma GPx is associated with semen quality since its activity is positively correlated with sperm concentration, motility, and morphology, and negatively with abnormal sperm. Therefore, the present findings may indicate that sperm cells from GF ejaculates receive better antioxidant protection from external sources, which increases their viability. Interestingly, a somewhat different relationship between antioxidant enzyme activities was noted in spermatozoa derived from GF and IF ejaculates. In mitochondria, SOD serves as the primary enzyme catalysing the removal of anions, and it cannot be substituted by other antioxidant enzymes [24]. Superoxide dismutase activity was higher in spermatozoa from IF ejaculates than in the extracellular environment, which could indicate that more superoxide anion radicals are generated inside cells and that SOD acts as a primary antioxidant enzyme. This assumption may be supported by the significantly higher Zn^2+^ content of spermatozoa from IF ejaculates, which enhanced the bioavailability of this microelement. Conversely, lower GPx and CAT activities in IF sperm cells may result in insufficient protection against hydrogen peroxide (H_2_O_2_). Superoxide dismutase activity generates this molecule, which induces lipid peroxidation in membranes rich in polyunsaturated fatty acids (PUFAs). This process produces lipid aldehydes that impair mitochondrial function and adenosine triphosphate (ATP) synthesis, ultimately reducing sperm motility and viability. H_2_O_2_ also compromises membrane integrity, disrupting transport and signal transduction required for capacitation [27]. Thus, lower GPx and CAT activities could have contributed to the accumulation of H_2_O_2_ and, consequently, diminished sperm viability in IF ejaculates. The presence of GSH in turkey semen has been previously confirmed [28]. In the present study, a significant difference in GSH content was observed between GF and IF semen. Notably, in IF ejaculates, GSH concentration was higher in the seminal plasma and lower in the spermatozoa. Some studies have reported a positive correlation between seminal plasma GSH concentration and improved sperm quality parameters [29]. However, elevated GSH levels in seminal plasma may also result from excessive ROS production by abnormal spermatozoa, which can stimulate thiol synthesis as a protective response against oxidative stress [29]. A similar compensatory mechanism may explain the higher GSH concentration observed in the seminal plasma of IF ejaculates. Our previous study on preserved turkey semen demonstrated that GSH levels may fluctuate with prolonged semen storage. Elevated GSH levels were associated with the need to combat intensified oxidative stress [28]. In the current study, lower GSH levels in spermatozoa from IF ejaculates may indicate the need for enhanced antioxidant protection, as a higher percentage of NO-generating spermatozoa and elevated MDA levels were also observed. In general, excessive production of ROS, including H_2_O_2_, superoxide radicals (O_2_^−^), NO, and lipid peroxidation products such as MDA or thiobarbituric acid reactive substances (TBARS), is associated with an increased risk of male infertility [30]. According to Bucak et al. [31], elevated MDA levels in semen are a major factor contributing to the deterioration of sperm quality, including reduced sperm motility. Malondialdehyde content was lower in GF ejaculates (*p* ≤ 0.05), which provides indirect evidence for the lower intensity of oxidative processes in semen and/or greater antioxidant capacity of the reproductive system.

### 3.2. Potential Impact of Proteome Composition on IF Semen

Numerous proteins in the ejaculate have been associated with male infertility or impaired fertility. These polypeptides may not only explain recurrent fertilisation failures, but may also serve as novel diagnostic tools for enriching conventional semen analyses [32]. Among these proteins, we can distinguish those that are associated with sperm motility and differentiation (e.g., lactate dehydrogenase, glyceraldehyde-3-phosphate dehydrogenase, triosephosphate isomerase, tubulin, and outer dense fibre protein 2); sperm-zona pellucida interaction and sperm-oolemma penetration (e.g., zona pellucida sperm-binding proteins, spermadhesins, IZUMO family members, and cysteine-rich secretory proteins); acrosome biogenesis and acrosome reaction (e.g., sperm acrosome membrane-associated proteins and seminal plasma secretory actin-binding proteins), nucleus composition (e.g., histones and protamines), as well as those formed by peripheral production (e.g., beta-defensin, semenogelin, fibronectin, lactoferrin) and post-translational modification (e.g., flagella calcium binding protein, heat shock-related proteins, and ADAM family members) [33].

This study revealed distinct proteomic profiles in the seminal plasma and spermatozoa of GF and IF ejaculates. The identified proteins likely represent metabolic pathways that influence semen fertility, either by promoting or mitigating ROS production. According to O’Flaherty [34], peroxiredoxins (PRDXs), especially PRDX6, play a key role in safeguarding spermatozoa against oxidative stress by regulating ROS generation. By acting as both a peroxidase and a calcium-independent phospholipase A2, PRDX6 is crucial for maintaining sperm viability and motility. Peroxidase activity is essential for eliminating H_2_O_2_, other hydroperoxides, and peroxynitrite (ONOO^−^), while calcium-independent phospholipase A_2_ (iPLA_2_) repairs oxidatively damaged membranes [35]. In mammals, this enzyme helps regulate ROS levels during the complex process of capacitation, thereby enabling the sperm to acquire fertilising capability [34]. Although capacitation is not required for successful fertilisation in birds, PRDX6 has been identified in chickens [36] and turkeys [10,11]. Spermatozoa lacking PRDX6 exhibit reduced viability and significantly lower motility compared to controls [37]. The presence of PRDX6 in several protein bands derived from seminal plasma and spermatozoa in GF turkey ejaculates may indicate higher antioxidant potential and an enhanced capacity to counteract the effects of ROS. In most animals, ferritin consists of two subunits: the ferritin heavy chain (FHC or FTH) and the ferritin light chain (FLC) [38]. Ferritin contains numerous polypeptide subunits and can store as many as 4500 iron atoms within its hollow spherical structure. In chickens, ferritin consists solely of the FHC subunit, which exhibits ferrous oxidase activity comparable to that of mammalian FHC [39]. The ferrous oxidase activity of FHC can prevent Fe^2+^ from producing ROS through the Haber–Weiss and Fenton reactions. Knockdown of the FHC in K562 cells disrupts the formation of protein disulphide bonds, leading to protein misfolding and elevated intracellular ROS levels [40]. The presence of ferritin in GF turkey seminal plasma may help limit ROS accumulation. However, ferritin may constitute only one element of the broader and more efficacious antioxidant response in turkey semen, and its effectiveness may be limited. Glyoxalase 2, encoded by the hydroxyacylglutathione hydrolase (HAGH) gene, is a member of the metallo-β-lactamase family and is found in both the mitochondria and cytoplasm. Hydroxyacylglutathione hydrolase helps detoxify methylglyoxal (MG) and may contribute to cell survival under metabolic stress. Recent research has shown that HAGH also plays other roles, especially in oxidative stress responses. During oxidative stress, Glo2 activation is essential; however, during redox homeostasis, it may be silenced without jeopardising cell viability. Glo2 may either release or utilise GSH to induce S-glutathionylation of target proteins, which indicates that the enzyme’s regulatory role can be enhanced under specific pathological conditions. Mitochondrial Glo2 contributes to the regulation of multiple processes, including apoptosis [41]. The activity of this enzyme depends on two divalent Zn^2+^ ions. Thus, their presence among the proteins identified in seminal plasma from GF ejaculates could be linked to its enhanced antioxidant capacity. It can also be assumed that a higher Zn^2+^ content in GF seminal plasma could help sustain Glo2 activity.

Enolases (EC 4.2.1.11) catalyse the conversion of 2-phosphoglycerate to phosphoenolpyruvate and occur in three eukaryotic isoforms: α (ENO1), γ (ENO2), and β (ENO3) [42]. The alpha-enolase (ENO1) variant has diverse roles beyond glycolysis, including stress response, infection, cancer progression, and reproduction [43]. The expression of ENO1 is altered during cell growth and in response to pathological conditions. Furthermore, changes in protein expression are influenced by both the duration of exposure and oxygen concentration. ENO1 has been reported to enhance anaerobic metabolism under hypoxic conditions, thereby contributing to cellular protection during oxygen deprivation [44]. Under anaerobic conditions, turkey sperm exhibit reduced glycolytic capacity, rapid ATP depletion, and decreased motility relative to aerobic conditions, which significantly impair their fertilising potential. The limited glycolytic ability of turkey sperm leads to a pronounced decline in ATP levels, ultimately compromising overall fertility [45]. These results could point to low oxygen availability in IF ejaculates. Given the higher sperm concentration, it can be assumed that not all spermatozoa had adequate access to oxygen, as highly motile sperm might have contributed to local oxygen depletion. Thus, the presence of enolase in IF semen may reflect insufficient oxygen in the environment. Interestingly, an acute hypoxic event can trigger a burst of superoxide production [46], and higher SOD activity in spermatozoa from IF ejaculates could be indicative of such an event. Research indicates that cells under heat stress or glucose deprivation often upregulate proteins such as heat shock proteins (HSPs) and glucose-regulated proteins, including alpha-enolase (ENO1), to support adaptation [47]. Glycolytic enzymes, particularly ENO1, are enriched on the membranes of apoptotic cells and considered representative SUPER proteins (surface-exposed, ubiquitously expressed, protease-sensitive, evolutionarily conserved, and typically present in viable cells) [48]. Therefore, the detection of ENO1 in IF ejaculates may reflect the presence of immature or abnormal sperm undergoing apoptosis. Additionally, ENO1 is associated with neutrophils and immune responses, suggesting that external stressors or local inflammation could contribute to its elevated expression. The enzyme is highly conserved, and its activity may be influenced by metal ions such as manganese and zinc [43]. Thus, higher amounts of Zn^2+^ in spermatozoa from IF ejaculates may have contributed to its activation. Sumarsono et al. [49] found that ENO1 activity in the plasma membrane of Bali cattle sperm was affected by sperm concentration, but it was not related to sperm quality. Therefore, it cannot be excluded that the presence of ENO1 in spermatozoa derived from IF semen may also be associated with their higher concentration. Beta-2-microglobulin (B2M) is a component of the MHC-I molecule in many species and may contribute to inter-species infertility and speciation [50]. Soluble B2M is widely present in bodily fluids, including serum, and is generally stable under physiological conditions, with elevated levels often reflecting increased immune activity [51]. In seminal plasma, B2M is normally produced in approximately equal amounts by the prostate and seminal vesicles. While a portion of B2M binds to spermatozoa, the free form is not readily incorporated [52]. Its relationship with sperm counts remains unclear: some studies suggest a positive association [53], whereas others report a decrease in B2M levels with increasing sperm counts [52]. Therefore, there is a possibility that the presence of B2M in IF ejaculates could result from inflammatory processes in the avian reproductive system or oxidative stress in semen. This assumption is supported by the presence of cytoplasmic aspartate aminotransferase (GOT1), mitochondrial aspartate aminotransferase (GOT2), L-lactate dehydrogenase A (LDHA) and L-lactate dehydrogenase B (LDHB) in some protein bands derived from IF semen. The release of the intracellular enzyme aspartate aminotransferase (GOT, also known as AST) into the extracellular space is indicative of sperm cell damage. This transaminase is predominantly localised in the midpiece of spermatozoa, whereas lactate dehydrogenase (LDH) is distributed in both the mitochondria and cytosol [54]. By reflecting plasma membrane stability, these enzymes are valuable indicators of semen quality. Their presence in IF semen may be indicative of a deterioration in its parameters. Fatty acid-binding proteins (FABPs) are a family of intracellular proteins essential for the transport and metabolism of fatty acids and other hydrophobic ligands, including saturated and unsaturated fatty acids, PUFAs, eicosanoids, and other lipids. FABPs are critical regulators of lipid metabolism, gene expression, and signal transduction, and serve as mediators of metabolic and inflammatory processes [55]. In humans, ten low-molecular-weight isoforms (14–15 kDa) have been identified [56]. B-FABP (FABP7) exhibits high affinity for n-3 PUFAs and is primarily expressed in astrocytes and oligodendrocytes. Although FABP7 is typically restricted to the embryonic brain [57], it has been detected in turkey semen [10]. At the molecular level, FABP7 facilitates fatty acid uptake and lipid droplet formation [57], which protects astrocytes against ROS toxicity. In turkeys, the presence of lipid droplets and altered lipid metabolism may contribute to sperm dysfunction and reduced fertilising capacity [58]. Therefore, the presence of this isoform in the semen of individuals with reduced fertility could be indicative of an inflammatory process, possibly caused by excessive ROS production.

Atikuzzaman et al. [59] demonstrated that higher fertility in domestic chickens is reflected in the seminal fluid proteome, and conserved proteins such as serum albumin and ovotransferrin were also detected in turkey semen. Certain proteins, including aspartate aminotransferase, annexin A5, and glutathione S-transferase 2, were associated with low-fertility ejaculates, whereas glyceraldehyde-3-phosphate dehydrogenase was unique to high-fertility samples. Overall, seminal fluid from low-fertility ejaculates contained fewer immune-related proteins and lower levels of the anti-inflammatory factor TGF-β2. Similarly, Carvalho et al. [60] reported an abundance of cilia- and flagella-associated protein 100, ENO1, and tubulin beta-7 chain in subfertile roosters. Proteins differentially expressed in fertile and subfertile chickens included metabolic enzymes, stress-related proteins, and structural proteins, several of which were also detected in turkeys. The results of the current study are partly consistent with these findings, suggesting that the presence and activity of specific proteins may vary among individuals and, ultimately, influence turkey fertility.

### 3.3. Potential Impact of Other Factors on IF Semen

In the present study, the effects of sanitary, nutritional, and health factors were ruled out; however, other variables may still contribute to reduced ejaculate quality. Notably, breeder age has been reported to significantly influence reproductive performance [61], with older flocks typically exhibiting lower fertility and hatchability than younger ones. However, the male turkeys examined in the present study had only recently reached maturity; therefore, the semen may not yet have reached its maximum fertilising capacity. In turkeys, fertility is influenced by age, and both young and older males potentially exhibit a lower fertility than mature, healthy toms. The age at which male turkeys reach maturity, as indicated by the onset of semen production, varies significantly [62]. Ejaculates were collected from turkeys at the onset of their reproductive life, and this factor could have contributed to the observed variation in ejaculate quality. Therefore, the influence of age on fertility cannot be completely ruled out. Adhikari et al. [63] found that egg production was higher in April, May, and June, and these months represent the peak egg-laying season in turkeys. In the present experiment, ejaculates were collected at the end of March; therefore, it can be assumed that not all individuals had entered the period of increased egg production.

These observations suggest that both the analysed semen parameters and semen proteome composition may have influenced the fertilisation indices. During cyclic decreases in fertilisation rates, the semen factor should be considered once other factors have been excluded.

## 4. Materials and Methods

### 4.1. Material Collection

The experimental material comprised 37 ejaculates collected from white turkeys of the Hybrid Converter^NOVO^ line (Hendrix Genetics BV, Boxmeer, The Netherlands). The birds were aged between 35 and 37 weeks and were housed on the GRELAVI farm (owned by Hendrix Genetics BV, Boxmeer, The Netherlands) in Komorowo (Warmian-Masurian Voivodeship, Poland). Semen was collected at the end of March 2023. Ejaculates were collected from males exhibiting suboptimal egg fertilisation rates ranging from 80% to 84%, whereas a fertilisation rate exceeding 88% was considered satisfactory.

On this commercial farm, AI is performed once a week following a standardised schedule: the initial three inseminations are conducted within the first ten days, and subsequent inseminations take place at one-week intervals. These procedures are initiated once the birds reach reproductive readiness, i.e., typically two weeks after the onset of photostimulation (approximately 14 ± 2 h of light per day) and the appearance of the first eggs. Fertilisation success is evaluated by egg candling on day 14 post-insemination. All eggs laid during the cycle are examined, which averages approximately 40,000 eggs per week on the studied farm. When fertilisation rates remain below the satisfactory threshold following two consecutive candling assessments conducted at weekly intervals, a comprehensive investigation of potential fertility-affecting factors is undertaken. The breeding females used in these procedures were between 31 and 33 weeks of age, and were at the peak of their laying cycle.

Following verification that environmental conditions, dietary composition, and flock health were uniform across all groups, the subsequent analyses focused on semen quality parameters in males exhibiting reduced fertilisation performance. The collected ejaculates were divided into two groups: impaired fertility (IF; *n* = 27) and good fertility (GF; *n* = 10). GF ejaculates were obtained from individuals that were housed in nearby buildings and consistently achieved fertilisation rates above 90% after egg laying, while the IF group included ejaculates with fertilisation rates below the 84% threshold. Ejaculates were collected by experienced farm workers using the manual massage method [64]. Semen from each male was collected only once. Farm personnel marked individuals after collection to prevent repeated sampling. All ejaculates were collected into separate syringes and immediately transported to the laboratory in a thermobox. Upon arrival, the samples were assessed for sperm concentration, motility, and viability. Next, they were centrifuged at 10,000× *g* for 10 min. The obtained seminal plasma was transferred to clean tubes, and the remaining spermatozoa precipitates were washed twice with 0.85% NaCl solution and centrifuged twice to remove any debris. Finally, TBS with 1% SDS (50 mM TRIS, 150 mM NaCl, 1% SDS; pH 7.5) was added to the resulting pellets, and sperm extracts were prepared. Samples were stored at −80 °C until subsequent analysis.

### 4.2. Assessment of Sperm Concentration

Total sperm counts in each ejaculate were determined using a light microscope (Olympus CH30, Olympus, Tokyo, Japan; 40× magnification) and a Bürker counting chamber (Equimed-Medical Instruments, Cracow, Poland). Before analysis, each sample was diluted with 0.85% NaCl solution to a final ratio of 1:800. The total sperm count was determined by counting cells in twenty squares of the Bürker chamber grid, and the average number of sperm cells per square was calculated.

### 4.3. Assessment of Sperm Motility

Sperm motility was evaluated using a computer-assisted semen analysis system (CASA; Hamilton Thorne IVOS, version 12.3, Beverley, MA, USA). A sperm motility buffer (50 mM TRIS, 120 mM NaCl, 10 mM glucose, 2 mM CaCl_2_, 0.5% BSA (pH = 7.4) was added to 30-μL sperm suspensions at a 1:1 ratio, and the mixtures were placed in a thermoblock (Thermo Block TDB-120, Biosan, Riga, Latvia) set to 38 °C for 10 min. Following incubation, 10-μL samples were placed in a Makler chamber (pre-warmed to 38 °C). According to the manual, special settings are required to analyse turkey semen: 60 frames were acquired at a frame rate of 60 Hz, with a minimum cell contrast of 35% and a minimum cell size of 5 pixels. The VAP threshold was set to 50 μm/s, the STR threshold to 80.0%, the VAP cutoff to 30.0 μm/s, and the VSL cutoff to 15.0 μm/s.

### 4.4. Assessment of Sperm Plasma Membrane Integrity (SYBR-14/PI)

The integrity of sperm plasma membranes in the head region was evaluated using the method developed by Garner and Johnson [65]. First, spermatozoa were diluted to a concentration of 30 × 10^6^ spermatozoa/mL using a HEPES/BSA buffer with the following composition: 10 mM HEPES, 130 mM NaCl, 4 mM KCl, 14 mM fructose, 1 mM CaCl_2_, 0.5 mM MgCl_2_, and 0.1% BSA (pH 7.4). Then, 1 μL of SYBR-14 dye was added to each diluted sample of 100 μL. The samples were incubated at 38 °C for 10 min. After incubation, 1 μL of propidium iodide (PI) was added to each sample, and the samples were incubated under the same conditions for an additional 10 min. Stained 10-μL samples were mounted on a standard slide and examined microscopically. Spermatozoa were assessed under a fluorescence microscope (Olympus CH 30 RF-200, Olympus, Tokyo, Japan) at 600× magnification. The heads of spermatozoa with intact plasma membranes fluoresced green after staining with SYBR-14, whereas those with damaged membranes fluoresced red after staining with PI. Spermatozoa were counted in at least 10 random fields of view. The results were expressed as the percentage of spermatozoa with intact plasma membranes (%).

### 4.5. Assessment of Sperm Mitochondrial Membrane Potential (JC-1/PI)

Two fluorescent dyes, JC-1 (iodide-5,5′, 6,6′-tetrachloro-1,1′,3,3′-tetraethylbenzimidazole-carbocanine) and PI, were used to assess sperm MMP. In the first step, semen samples were diluted to a concentration of 30 × 10^6^ sperm/mL in HEPES/BSA buffer. Then, 0.33 μL of JC-1 solution was added to each sample and incubated for 10 min at 38 °C. After incubation, 1 μL of PI solution was added to each sample, and the samples were observed under a fluorescence microscope. Spermatozoa were evaluated under a fluorescence microscope (Olympus CH 30 RF-200, Olympus, Tokyo, Japan) at 600× magnification. At least 200 spermatozoa were evaluated in random fields of view in each sample. Spermatozoa exhibiting orange fluorescence in the midpiece were considered to have high MMP (active mitochondria, JC-1^+^/PI^−^), whereas spermatozoa exhibiting green fluorescence in the midpiece region and/or red fluorescence in the head region were classified as non-viable cells with low MMP (JC-1^−^/PI^+^). The results were presented as the percentage of spermatozoa with high MMP (%).

### 4.6. Assessment of Nitric Oxide (NO)-Generating Spermatozoa (DAF-2DA)

Nitric oxide production was evaluated using the DAF-2DA staining method described by Lampiao et al. [66]. Nitric oxide-producing sperm were assessed using the DAF-2DA (4,5-diaminofluorescein diacetate) fluorescent dye. Each sample was first diluted with HEPES/BSA buffer to a final concentration of 30 × 10^6^ spermatozoa/mL. Then, 100 μL of a 10 μM DAF-2DA solution in PBS was added to each sample. The samples were incubated for 120 min in a thermoblock (Thermo Block TDB-120, Biosan, Riga, Latvia) at 39 °C in the dark. Nitric oxide-generating spermatozoa were counted under a fluorescence microscope (Olympus CH 30 RF-200, Olympus, Tokyo, Japan) equipped with a DMB filter (blue light). The results were presented as the percentage of spermatozoa (%) showing green-blue fluorescence relative to the total number of spermatozoa previously counted in the same fields of view under bright light.

### 4.7. Assessment of Antioxidant System Efficiency

#### 4.7.1. Antioxidant Enzyme Triad

Antioxidant enzyme activity was measured in both seminal plasma and spermatozoa. Enzyme activity in seminal plasma was expressed in international units per millilitre (U/mL) or micromoles per millilitre per minute (µM/mL/min). Enzyme activity in spermatozoa was adjusted based on sperm concentrations (U/10^9^ sperm).

##### Determination of Superoxide Dismutase (SOD) Activity

Superoxide dismutase activity was determined using the RANSOD Kit (Randox Laboratories, Crumlin, UK) according to the manufacturer’s instructions, and was measured at 505 nm. One unit of SOD activity was defined as the amount of the enzyme causing 50% inhibition of iodonitrotetrazolium chloride (INT) reduction at 37 °C and pH 7.0.

##### Determination of Glutathione Peroxidase (GPx) Activity

Glutathione peroxidase activity was determined using the RANSEL Kit (Randox Laboratories, Crumlin, UK) according to the manufacturer’s protocol and was measured at 340 nm. One unit of GPx catalyses the oxidation of 1.0 µM GSH to GSSG per minute at 25 °C, pH 7.0, in the presence of H_2_O_2_, leading to a simultaneous oxidation of NADPH to NADP^+^ and a corresponding decrease in absorbance.

##### Determination of Catalase (CAT) Activity

Catalase activity was measured using a commercial Catalase Assay Kit (Sigma-Aldrich, Merck, Burlington, MA, USA) based on the manufacturer’s instructions and was assessed at 520 nm. One unit of CAT decomposes 1 M H_2_O_2_ per minute at a substrate concentration of 50 mM H_2_O_2_ at 25 °C (pH 7.0).

#### 4.7.2. Antioxidant Agents

The content of antioxidants was measured in both seminal plasma and spermatozoa. Their concentration in seminal plasma was expressed in moles per millilitre (M/mL) or micrograms per millilitre (µg/mL). Their content in spermatozoa was converted to sperm concentration (×10^9^ sperm).

##### Determination of Glutathione (GSH) Content

Glutathione content was determined using the Bioxytech GSH-400 Kit from Bio-Sciences (Burlingame, CA, USA) according to the manufacturer’s manual. The concentration of GSH (reduced glutathione) + GSSG (oxidised glutathione) was determined by measuring the change in absorbance at 400 nm at 25 °C.

##### Determination of Zinc (Zn^2+^) Content

Zinc concentration was determined according to the method described by Lampugnani and Maccheroni [67]. Following deproteinisation, zinc levels in seminal plasma and spermatozoa extracts were measured colourimetrically using 4-(2-pyridylazo)resorcinol sodium salt (PAR-Na) as a chromogenic reagent. The zinc–PAR complex was quantified at a wavelength of 490 nm, at a temperature of 37 °C and pH 9.5. The results were expressed in µg/mL for seminal plasma and µg per 10^9^ spermatozoa mL^−1^ for sperm samples.

### 4.8. Assessment of Oxidative Damage Expressed as Malondialdehyde (MDA) Content

Malondialdehyde content was determined using the Bioxytech MDA-586 Kit from AOXRE Bio-Sciences (Burlingame, CA, USA), following the manufacturer’s instructions. In the MDA-586 assay, the MDA content of a sample is calculated based on probe absorbance at 586 nm and a standard curve.

### 4.9. Analysis of Turkey Semen Protein Profiles by Two Separation Methods

Before separation, the total protein content in all probes was measured using the Bradford reagent (Sigma-Aldrich, Merck, Burlington, MA, USA), following the manufacturer’s specified procedure.

#### 4.9.1. SDS-PAGE Electrophoresis

Proteins of turkey seminal plasma and spermatozoa were separated in 12% polyacrylamide gels using a buffer composed of 50 mM Tris, 250 mM glycine, and 0.5% SDS (pH 8.3). The separation was conducted in a Mini-Protean II Cell apparatus (Bio-Rad, Hercules, CA, USA) based on the method proposed by Laemmli [68]. Next, 5 μL of concentrated lysis buffer (1 M Tris-HCl, 20% SDS, 2% glycerol, 2% β-mercaptoethanol, 2% bromophenol blue; pH = 6.8) was added to samples with a standardised protein concentration (1.5 mg/mL). The samples were then heated at 95 °C for 5 min in a Thermo Block TDB-120 thermostat (Biosan, Riga, Latvia). From each prepared sample, 15 μL was loaded into individual wells of a polyacrylamide gel and separated electrophoretically under constant current. The separation was performed at 80 V for the initial 20 min, then at 130 V for the remainder. Precision Plus Protein Standards (Bio-Rad, Hercules, CA, USA) were used in the procedure (molecular weight range: 250–10 kDa). The destained gels were analysed densitometrically using Multi-Analyst software (version 1.1; Bio-Rad Laboratories, Hercules, CA, USA).

#### 4.9.2. TRICINE-PAGE Electrophoresis

This method allowed for more precise separation of proteins with a molecular weight below 70 kDa. A three-layer polyacrylamide gel with different densities was used, consisting of a stacking layer (4%), an intermediate layer (10%), and a separating layer (15%). Successive layers were poured into the gel wells at roughly 15 min intervals and overlaid with 100 μL of distilled water to thoroughly polymerise each layer. After polymerisation, the water was removed with a syringe, and another gel layer was applied. Two buffers were used during separation: a cathodic buffer (0.1 M Tris, 0.1 M Tricine, 0.1% SDS) and an anodic buffer (0.2 M Tris). The separation was carried out at 80 V for the initial 20 min and at 130 V for the remainder of the process. Precision Plus Protein Standards (Bio-Rad, Hercules, CA, USA) were used as the reference standards (molecular weight range from 250 to 10 kDa). The destained gels were analysed densitometrically using Multi-Analyst software (version 1.1; Bio-Rad Laboratories, Hercules, CA, USA).

### 4.10. Identification of Selected Proteins by Nano LC-MS/MS Mass Spectrometry

Gel sections were excised and subjected to decolourisation, reduction, alkylation, and digestion procedures. Bands were first incubated in 50 μL of 100 mM ammonium carbonate solution for 10 min with shaking in a thermoblock. Acetonitrile (50 μL) was then added, and the mixture was shaken at 600 rpm for 10 min. The supernatant was removed, and gel fragments were dehydrated with 100% acetonitrile, followed by shaking under the same conditions. Next, gel fragments were treated with 50 μL of aqueous dithiothreitol (DTT) solution and reduced at 90 °C for 10 min. Then, 50 μL of 5 mM iodoacetamide in 100 mM ammonium carbonate buffer was added for alkylation, carried out at 90 °C for 10 min, with shaking at 600 rpm. After alkylation, the iodoacetamide solution was removed, and the gel was dehydrated again with acetonitrile. Subsequently, 1.5 pmol of trypsin (Promega, Madison, WI, USA) was added, and the mixture was rehydrated for 5 min. The material was incubated overnight in carbonate buffer at 37 °C in a thermoblock. The following day, peptides were extracted using 50 mM carbonate buffer, followed by two extractions with 5% formic acid solution in 50% acetonitrile. Each extraction step lasted 15 min at 37 °C. Peptides eluted from all fractions were combined, lyophilised, and dissolved in 30 μL of a 4% acetonitrile solution with 0.1% formic acid. Nano LC-MS/MS analysis was conducted using an Ultimate 3000 HPLC/UPLC system (Thermo Fisher Scientific, Waltham, MA, USA) connected to an Exploris 240 mass spectrometer (Thermo Fisher Scientific, Waltham, MA, USA) in positive ion mode. A sample of 5 μL was injected into the system, comprising an RP C18 precolumn (0.3 × 5 mm, 5 μm particle size) and an RP C18 column (15 cm, 3 μm, 75 μm particle size) (Thermo Fisher Scientific, Waltham, MA, USA). The mobile phase flow rate was 300 nL/min, and samples were eluted using a 33 min gradient of solutions A (0.1% fluoroacetic acid in water) and B (0.1% fluoroacetic acid in acetonitrile). The gradient was as follows: 8% solution B at 0 and 4 min, 35% at 30 min, 90% at 30 min 30 s, then 31 min 30 s, and back to 8% at 32 and 33 min. Raw data were processed with Data Analysis software (version 4.0, Bruker-Daltonics, Germany) to generate mgf files for protein identification using Mascot 3.0 software (Matrix Science, London, UK). All mgf files were searched against the UniProtKB database for the animal taxonomic group, with emphasis on the following poultry species: *Meleagris gallopavo*, *Gallus gallus*, *Coturnix japonica*, *Phasianus colchicus*, and *Numida meleagris*. Only protein identifications with Mascot scores ≥ 90 were included in the results.

### 4.11. Functional Analysis of Identified Proteins

The proteome composition of seminal plasma and spermatozoa obtained from GF and IF turkey semen was compared using a free web tool for creating Venn diagrams (https://www.biotools.fr/misc/venny, accessed on 5 August 2025).

Functional enrichment of proteins from GF and IF turkey semen was analysed based on the associated biological processes using the PANTHER Classification System v. 19.0 (Gene Ontology (GO) online tool, http://pantherdb.org, accessed on 25 August 2025). Molecular function was estimated by applying Fisher’s exact test with the false discovery rate (FDR) correction on the PAN-GO Human Functionome platform. Only statistically significant terms (FDR ≤ 0.05) were included in the manuscript. All data were retrieved from the reference database that included the targeted poultry species. When protein sequences for the targeted species were unavailable, the *Homo sapiens* database was searched as a comprehensive and regularly updated reference.

### 4.12. Statistical Analysis

The results were processed in the Statistica programme (version 13.3., StatSoft, TIBCO Software, Palo Alto, CA, USA) and presented as arithmetic means and standard deviations (SDs). All parameters were analysed using the Student’s t-test for independent samples. The residuals from each model were analysed to verify the assumptions of homogeneity of variance and normality. The variables that deviated from a normal distribution were appropriately transformed. The magnitude of differences was assessed at two significance levels: *p* ≤ 0.001 and *p* ≤ 0.05. Differences in the optical density (OD) of individual fractions were calculated from all electropherograms corresponding to each ejaculate component and semen type. A non-parametric analysis and the Mann–Whitney U test were used for this purpose.

## 5. Conclusions

Impaired male fertility is often associated with oxidative stress in semen. The results of this study indicate a disruption of redox homeostasis in IF ejaculates, evidenced by reduced total sperm motility and plasma membrane integrity, assessed through fluorescent dyes, as well as significant variations in SOD, GPx and CAT activities and MDA level in both seminal plasma and spermatozoa. These observations were further substantiated by proteomic profiling, which revealed differences in the expression of redox- and metabolism-associated proteins, including the exclusive presence of PRDX6 in GF semen, and of ENO1, FABP7, GOT1, and LDHB in IF samples. Collectively, these biochemical and proteomic changes indicate that excessive production of ROS and altered antioxidant defence pathways may negatively impact semen quality and fertilising potential in turkeys. The current findings offer indirect evidence that analysing semen parameters in turkeys is crucial for optimising industrial breeding practices, as biochemical and biological assessments of semen are essential for understanding poultry reproductive function and improving breeding outcomes.

## Figures and Tables

**Table 1:** The quality parameters of spermatozoa from turkey ejaculates with good fertility (GF) and impaired fertility (IF).

Parameters	GF	IF	*p*-Value
Sperm concentration (×10^9^)	4.7 ± 1.0 *	5.6 ± 1.7 *	0.022
PMI (%)	88.4 ± 4.6 **	78.4 ± 6.8 **	0.000
MMP (%)	79.7 ± 6.2	79.9 ± 7.9	0.637
NO (%)	29.3 ± 11.2 *	42.4 ± 19.0 *	0.044
TMOT (%)	81.2 ± 6.6 *	71.7 ± 8.5 *	0.002
PMOT (%)	49.7 ± 13.2	46.3 ± 24.6	0.672
VAP (µm/s)	74.0 ± 3.7	80.3 ± 13.3	0.148
VSL (µm/s)	70.7 ± 5.4	73.9 ± 15.0	0.209
VCL (µm/s)	116.8 ± 8.9 *	105.9 ± 14.4 *	0.028
ALH (µm)	3.59 ± 0.5	3.29 ± 0.6	0.146
BCF (Hz)	22.4 ±8.3	18.7 ± 6.8	0.150
STR (%)	81.6 ± 6.0 **	90.3 ± 5.3 **	0.000
LIN (%)	66.7 ± 6.3	71.7 ± 8.5	0.125

Values marked with ** are highly statistically significant (*p* ≤ 0.001), and values marked with * are significant (*p* ≤ 0.05). GF—good fertility ejaculates, IF—impaired fertility ejaculates PMI—plasma membrane integrity, MMP—mitochondrial membrane potential, NO—percentage of sperm generating nitric oxide, TMOT—total motility, PMOT—progressive motility, VAP—average path velocity, VSL—straight line rectilinear velocity, VCL—curvilinear velocity, ALH—amplitude of lateral head displacement, BCF—beat-cross frequency, STR—straightness, LIN—linearity coefficient.

**Table 2:** The antioxidant potential of seminal plasma in turkey ejaculates with good fertility (GF) and impaired fertility (IF).

Parameters	GF	IF	*p*-Value
Total protein (mg/mL)	7.9 ± 2.0 **	5.6 ± 1.4 **	0.000
SOD activity (U/mL)	2.4 ± 0.6 **	1.1 ± 0.4 **	0.000
GPx activity (U/mL)	0.4 ± 0.1 **	0.2 ± 0.1 **	0.001
CAT activity (µM/min/mL)	14.4 ± 10.3 *	39.2 ± 29.9 *	0.014
GSH (M/mL)	0.5 ± 0.02 **	0.9 ± 0.03 **	0.000
MDA (µM/mL)	11.5 ± 11.7 **	28.4 ± 12.2 **	0.000
Zn^2+^ (µg/mL)	151.9 ± 58.9 **	49.7 ± 24.6 **	0.000

Values marked with ** are highly statistically significant (*p* ≤ 0.001), and values marked with * are significant (*p* ≤ 0.05). GF—good fertility ejaculates, IF—impaired fertility ejaculates SOD—superoxide dismutase, GPx—glutathione peroxidase, CAT—catalase, GSH—glutathione, MDA—malondialdehyde, Zn^2+^—zinc.

**Table 3:** The antioxidant potential of spermatozoa from turkey ejaculates with good fertility (GF) and impaired fertility (IF).

Parameters	GF	IF	*p*-Value
SOD activity (U/mL)	0.6 ± 0.2 **	1.2 ± 0.5 **	0.001
GPx activity (U/mL)	0.4 ± 0.1 **	0.2 ± 0.2 **	0.000
CAT activity (µM/min/mL)	114.3 ± 59.4 *	70.1 ± 44.5 *	0.012
GSH (M/10^9^ sperm)	0.2 ± 0.08 **	0.1 ± 0.07 **	0.000
MDA (µM/10^9^ sperm)	6.1 ± 4.3 **	14.8 ± 6.1 **	0.000
Zn^2+^ (µg/10^9^ sperm)	43.6 ± 12.1 *	92.2 ± 64.3 *	0.018

Values marked with ** are highly statistically significant (*p* ≤ 0.001), and values marked with * are significant (*p* ≤ 0.05). GF—good fertility ejaculates, IF—impaired fertility ejaculates. SOD—superoxide dismutase, GPx—glutathione peroxidase, CAT—catalase, GSH—glutathione, MDA—malondialdehyde, Zn^2+^—zinc.

**Table 4:** Mean optical density (OD) of turkey seminal plasma fractions selected for protein identification.

Protein [kDa]	GF	IF
107	0.28 ± 0.02	0.37 ± 0.03
80	0.21 ± 0.01	0.23 ± 0.01
49	0.30 ± 0.02	0.35 ± 0.01
41	0.45 ± 0.01	0.49 ± 0.01
29	0.30 ± 0.03	0.41 ± 0.02
26	0.28 ± 0.01	0.19 ± 0.02
25	0.39 ± 0.01	0.33 ± 0.01
18	0.35 ± 0.03	0.29 ± 0.04
16	0.27 ± 0.02	0.25 ± 0.02
12	0.17 ± 0.01	0.20 ± 0.01

GF—good fertility ejaculates, IF—impaired fertility ejaculates.

**Table 5:** Mean optical density (OD) of turkey spermatozoa fractions selected for protein identification.

Protein [kDa]	GF	IF
107	0.17 ± 0.03	0.24 ± 0.01
52	0.16 ± 0.01	0.19 ± 0.01
49	0.45 ± 0.03	0.68 ± 0.01
41	0.33 ± 0.02	0.59 ± 0.01
29	0.17 ± 0.01	0.29 ± 0.03
25	0.22 ± 0.01	0.19 ± 0.01
18	0.18 ± 0.02	0.16 ± 0.02
16	0.20 ± 0.01	0.26 ± 0.02

GF—good fertility ejaculates, IF—impaired fertility ejaculates.

## Data Availability

The original contributions presented in this study are included in the article and the Supplementary Materials. Further inquiries can be directed to the corresponding author.

## Supplementary Materials

The following supporting information can be downloaded at: https://www.mdpi.com/article/10.3390/ijms262211000/s1.
