# Supplementary material for: Can the Quality of Semen Affect the Fertilisation Indices of Turkey Eggs?
Source: Int J Mol Sci. 2025 Nov 13;26(22):11000. doi: 10.3390/ijms262211000 (PMC12652907; doi:10.3390/ijms262211000)
Supplement: Supplementary file 1 [file ijms-26-11000-s001.zip › ijms-3932743-supplementary.pdf]

Table S1. Proteins identified by SDS-PAGE and nano LC-MS/MS in seminal plasma derived from good fertility (GF) ejaculates.

Molecular weight [kDa]	Identified protein	Gene	Species	Molecular weight (Mascot) [Da]	No. of matched peptides	Sequence coverage [%]	Score
26	Albumin	*ALB*	*Gallus gallus*	71868	20	31	974
	Phosphoglycerate mutase 1	*PGAM1*	*Gallus gallus*	29051	16	24	807
	Creatine kinase B-type	*CKB*	*Gallus gallus*	43129	13	17	681
	Acrosin	*ACR*	*Meleagris gallopavo*	38724	12	16	589
	14-3-3 protein zeta	*YWHAZ*	*Gallus gallus*	27929	13	13	561
	14-3-3 protein theta	*YWHAQ*	*Gallus gallus*	28050	10	10	436
	Astacin-like metalloendopeptidase	*ASTL*	*Gallus gallus*	46929	12	9	404
	14-3-3 protein epsilon	*YWHAE*	*Gallus gallus*	29326	8	9	400
	Gelsolin	*GSN*	*Gallus gallus*	86120	8	8	369
	Peroxiredoxin-6	*PRDX6*	*Gallus gallus*	25075	12	7	351
	Sulfhydryl oxidase 1	*QSOX1*	*Gallus gallus*	83939	8	11	312
	Clusterin	*CLU*	*Coturnix japonica*	52395	3	8	266
	Hydroxyacylglutathione hydrolase, mitochondrial	*HAGH*	*Gallus gallus*	34545	5	6	235
	14-3-3 protein gamma	*YWHAG*	*Gallus gallus*	28384	4	6	209
	SPARC protein	*SPARC*	*Gallus gallus*	34894	3	5	164
	Ovotransferrin	*TF*	*Gallus gallus*	79551	3	5	161
	14-3-3 protein beta/alpha	*YWHAB*	*Gallus gallus*	28004	3	4	131
	Collagen alpha-1(XII) chain	*COL12A1*	*Gallus gallus*	341740	3	3	121
	Tubulin beta-3 chain	*TBB3*	*Gallus gallus*	50285	5	2	120
	Tubulin beta-7 chain	*TBB7*	*Gallus gallus*	50095	5	2	120
	Glyceraldehyde-3-phosphate dehydrogenase	*GAPDH*	*Gallus gallus*	35909	2	1	96
	1-phosphatidylinositol 4,5-bisphosphate phosphodiesterase zeta-1	*PLCZ1*	*Gallus gallus*	73285	2	1	94
25	Albumin	*ALB*	*Gallus gallus*	71868	24	38	1123
	Acrosin	*ACR*	*Meleagris gallopavo*	38724	12	27	748
	Creatine kinase B-type	*CKB*	*Gallus gallus*	43129	14	21	670

25	Triosephosphate isomerase	*TPI1*	*Gallus gallus*	26832	11	18	499
	Actin, cytoplasmic 2	*ACTG1*	*Gallus gallus*	42108	9	13	394
	Actin, cytoplasmic type 5	*ACT5*	*Gallus gallus*	42151	9	13	394
	Ras-related protein Rab-5B	*RAB5B*	*Gallus gallus*	23828	8	10	323
	Ras-related protein Rab-5C	*RAB5C*	*Gallus gallus*	23767	8	9	304
	Clusterin	*CLU*	*Coturnix japonica*	52395	4	9	300
	Peroxiredoxin-6	*PRDX6*	*Gallus gallus*	25075	9	12	272
	Ras-related protein Rab-10	*RAB10*	*Gallus gallus*	22763	6	11	257
	Phosphoglycerate mutase 1	*PGAM1*	*Gallus gallus*	29051	5	8	252
	Sulfhydryl oxidase 1	*QSOX1*	*Gallus gallus*	83939	4	9	202
	Collagen alpha-1(XII) chain	*COL12A1*	*Gallus gallus*	341740	5	9	200
	Apolipoprotein A-I	*APOA1*	*Gallus gallus*	30661	9	6	196
	Tubulin alpha-1 chain	*TUBA1*	*Gallus gallus*	46385	5	5	194
	14-3-3 protein zeta	*YWHAZ*	*Gallus gallus*	27929	4	8	168
	Mitochondria-eating protein	*SPATA18*	*Gallus gallus*	54997	2	9	166
	Astacin-like metalloendopeptidase	*ASTL*	*Gallus gallus*	46929	4	9	160
	Ferritin heavy chain	*FTH*	*Gallus gallus*	21249	5	8	159
	Hypoxanthine-guanine phosphoribosyltransferase	*HPRT1*	*Gallus gallus*	24878	3	7	146
	Carbonic anhydrase 2	*CA2*	*Gallus gallus*	29388	3	6	134
	Translin	*TSN*	*Gallus gallus*	26002	2	11	132
	Hydroxyacylglutathione hydrolase, mitochondrial	*HAGH*	*Gallus gallus*	34545	2	5	123
	Glyceraldehyde-3-phosphate dehydrogenase	*GAPDH*	*Gallus gallus*	35909	2	4	122
	Adenylate kinase isoenzyme 1	*AK1*	*Gallus gallus*	21783	2	4	106
	Tubulin beta-1 chain	*TBB1*	*Gallus gallus*	50333	3	3	102
	Tubulin beta-2 chain	*TBB2*	*Gallus gallus*	50377	3	3	102
	Tubulin beta-3 chain	*TBB3*	*Gallus gallus*	50285	3	3	102
	Tubulin beta-4 chain	*TBB4*	*Gallus gallus*	50844	3	3	102
	Tubulin beta-5 chain	*TBB5*	*Gallus gallus*	50395	3	3	102
	Tubulin beta-6 chain	*TBB6*	*Gallus gallus*	50692	3	3	102
	Tubulin beta-7 chain	*TBB7*	*Gallus gallus*	50095	3	3	102
	Protein-L-isoaspartate(D-aspartate) O-methyltransferase	*PCMT1*	*Gallus gallus*	24790	1	1	100
18	Albumin	*ALB*	*Gallus gallus*	71868	20	45	1810

18	Tubulin beta-3 chain	*TBB3*	*Gallus gallus*	50285	21	37	1507
	Creatine kinase B-type	*CKB*	*Gallus gallus*	43129	19	32	1321
	Tubulin beta-5 chain	*TBB5*	*Gallus gallus*	50395	15	23	1232
	Acrosin	*ACR*	*Meleagris gallopavo*	38724	12	19	917
	Tubulin beta-4 chain	*TBB4*	*Gallus gallus*	50844	10	17	799
	Creatine kinase S-type, mitochondrial	*CKMT2*	*Gallus gallus*	47510	9	15	687
	Voltage-dependent anion-selective channel protein 2	*VDAC2*	*Meleagris gallopavo*	30162	9	14	545
	Tubulin alpha-4 chain	*TBA4*	*Gallus gallus*	36483	9	12	465
	Cytochrome b-c1 complex subunit Rieske, mitochondrial	*UQCRFS1*	*Gallus gallus*	29710	7	11	395
	Cilia- and flagella-associated protein 20	*CFAP20*	*Gallus gallus*	22891	6	11	298
	Ubiquitin-ribosomal protein eS31 fusion protein	*RPS27A*	*Gallus gallus*	18310	6	9	237
	Polyubiquitin-B	*UBB*	*Gallus gallus*	34348	6	9	237
	Ferritin heavy chain	*FTH*	*Gallus gallus*	21249	5	8	199
	Succinate dehydrogenase [ubiquinone] iron-sulphur subunit	*SDHB*	*Gallus gallus*	33374	5	5	194
	Retinol-binding protein 4	*RBP4*	*Gallus gallus*	22843	4	4	162
	Lysozyme g	*LYZ*	*Gallus gallus*	23565	3	4	132
	Ovotransferrin	*TF*	*Gallus gallus*	79551	3	3	101
	Zona pellucida-binding protein 1	*ZPBP1*	*Gallus gallus*	36910	4	2	99
	T-complex protein 1 subunit theta	*CCT8*	*Gallus gallus*	60017	2	1	94
16	Albumin	*ALB*	*Gallus gallus*	71868	52	60	1967
	Ovotransferrin	*TF*	*Gallus gallus*	79551	21	45	891
	Creatine kinase B-type	*CKB*	*Gallus gallus*	43129	18	44	881
	Acrosin	*ACR*	*Meleagris gallopavo*	38724	15	43	594
	Astacin-like metalloendopeptidase	*ASTL*	*Gallus gallus*	46929	15	41	519
	Gelsolin	*GSN*	*Gallus gallus*	86120	10	39	431
	Sulfhydryl oxidase 1	*QSOX1*	*Gallus gallus*	83939	12	34	405
	Ferritin heavy chain	*FTH*	*Gallus gallus*	21249	16	27	397
	Tubulin beta-4 chain	*TBB4*	*Gallus gallus*	50844	15	24	382
	Apolipoprotein A-I	*APOA1*	*Gallus gallus*	30661	14	18	358
	Tubulin beta-5 chain	*TBB5*	*Gallus gallus*	50395	12	15	308
	1-phosphatidylinositol 4,5-bisphosphate phosphodiesterase zeta-1	*PLCZ1*	*Gallus gallus*	73285	7	14	252

16	Peptidyl-prolyl cis-trans isomerase B	*PPIB*	*Gallus gallus*	22456	7	11	232
	Transthyretin	*TTR*	*Gallus gallus*	16356	7	13	204
	Heat shock protein HSP 90-alpha	*HSP90AA1*	*Gallus gallus*	84406	6	10	192
	Heat shock cognate 71 kDa protein	*HSPA8*	*Gallus gallus*	71011	6	9	187
	Proteasome subunit beta type-5	*PSMB5*	*Gallus gallus*	27256	4	7	159
	Collagen alpha-1(XII) chain	*COL12A1*	*Gallus gallus*	341740	3	7	156
	Heat shock 70 kDa protein	*HSP70*	*Gallus gallus*	69936	6	5	172
	Actin, cytoplasmic 1	*ACTB*	*Gallus gallus*	42052	2	2	116
	Actin, cytoplasmic 2	*ACTG2*	*Gallus gallus*	42108	2	2	116
	Actin, cytoplasmic type 5	*ACT5*	*Gallus gallus*	42151	2	2	116
	Glyceraldehyde-3-phosphate dehydrogenase	*GAPDH*	*Gallus gallus*	35909	2	3	104
	Peroxiredoxin-6	*PRDX6*	*Gallus gallus*	25075	4	2	100

Table S2. Proteins identified by SDS-PAGE and nano LC-MS/MS in seminal plasma derived from impaired fertility (IF) ejaculates.

Molecular weight [kDa]	Identified protein	Gene	Species	Molecular weight (Mascot) [Da]	No. of matched peptides	Sequence coverage [%]	Score
107	Albumin	*ALB*	*Gallus gallus*	71868	39	41	1575
	Aminopeptidase Ey	*ANPEP*	*Gallus gallus*	109406	38	35	1555
	Collagen alpha-1(XII) chain	*COL12A1*	*Gallus gallus*	341740	27	32	1057
	Alpha-enolase	*ENO1*	*Gallus gallus*	47617	23	31	870
	Aspartate aminotransferase, cytoplasmic	*GOT1*	*Gallus gallus*	46134	12	19	556
	Creatine kinase B-type	*CKB*	*Gallus gallus*	43129	8	12	440
	Clusterin	*CLU*	*Coturnix japonica*	52395	4	9	275
	L-lactate dehydrogenase B	*LDHB*	*Gallus gallus*	36694	8	4	265
	Ovotransferrin	*TF*	*Gallus gallus*	79551	5	6	259
	NEL protein	*NEL*	*Gallus gallus*	96096	5	5	246
	Ovotransferrin	*TF*	*Gallus gallus*	79551	6	4	236
	Gelsolin	*GSN*	*Gallus gallus*	86120	5	4	234
	Heat shock cognate 71 kDa protein	*HSPA8*	*Gallus gallus*	71011	6	3	224
	Astacin-like metalloendopeptidase	*ASTL*	*Gallus gallus*	46929	5	3	217
	Fibrinogen beta chain	*FGB*	*Gallus gallus*	53272	4	3	194
	Transforming growth factor beta-2 proprotein	*TGFB2*	*Gallus gallus*	48431	3	8	182
	Neuronal growth regulator 1	*NEGR1*	*Gallus gallus*	38434	4	2	179
	Nucleoside diphosphate kinase	*NDK*	*Gallus gallus*	17448	2	2	137
	Translin	*TSN*	*Gallus gallus*	26002	7	1	131
	Acrosin	*ACR*	*Meleagris gallopavo*	38724	2	2	91
	Lysosome-associated membrane glycoprotein 1	*LAMP1*	*Gallus gallus*	45097	2	2	91
80	Albumin	*ALB*	*Gallus gallus*	71868	32	39	1384
	Collagen alpha-1(XII) chain	*COL12A1*	*Gallus gallus*	341740	17	22	677
	Cytoplasmic aconitate hydratase	*ACO1*	*Gallus gallus*	98639	11	18	551
	Heat shock protein HSP 90-alpha	*HSP90AA1*	*Gallus gallus*	84406	13	17	455
	Creatine kinase B-type	*CKB*	*Gallus gallus*	43129	8	12	387
	Elongation factor 2	*EEF2*	*Gallus gallus*	96343	10	9	358
	Alpha-actinin-4	*ACTN4*	*Gallus gallus*	104712	8	8	323

80	Endoplasmin	*HSP90B1*	*Gallus gallus*	91726	9	8	305
	Sulfhydryl oxidase 1	*QSOX1*	*Gallus gallus*	83939	8	6	299
	Ovotransferrin	*TF*	*Gallus gallus*	79551	7	5	271
	Acrosin	*ACR*	*Meleagris gallopavo*	38724	3	5	226
	Ubiquitin-ribosomal protein eS31 fusion protein	*RPS27A*	*Gallus gallus*	18310	2	3	128
	Polyubiquitin-B	*UBB*	*Gallus gallus*	34348	2	3	128
	Laminin subunit beta-1	*LAMB1*	*Gallus gallus*	35775	1	1	95
49	Alpha-enolase	*ENO1*	*Gallus gallus*	47617	68	42	2660
	Albumin	*ALB*	*Gallus gallus*	71868	32	31	1497
	Creatine kinase B-type	*CKB*	*Gallus gallus*	43129	27	28	1082
	Gamma-enolase	*ENO2*	*Gallus gallus*	47621	17	23	777
	Tubulin alpha-1 chain	*TUBA1*	*Gallus gallus*	46385	18	17	674
	Tubulin beta-4 chain	*TBB4*	*Gallus gallus*	50844	15	16	563
	Tubulin beta-5 chain	*TBB5*	*Gallus gallus*	50395	15	16	494
	ATP synthase subunit beta, mitochondrial	*ATP5B*	*Gallus gallus*	56650	15	10	488
	Tubulin alpha-5 chain	*TBA5*	*Gallus gallus*	50715	10	12	446
	Ovotransferrin	*TF*	*Gallus gallus*	79551	8	11	404
	Elongation factor 1-alpha	*EEF1A*	*Gallus gallus*	50467	10	9	285
	Eukaryotic initiation factor 4A-II	*EIF4A2*	*Gallus gallus*	46570	6	8	225
	Bleomycin hydrolase	*BLMH*	*Gallus gallus*	53397	5	7	221
	$Na^{(+)}/H^{(+)}$ exchange regulatory cofactor NHE-RF1	*NHERF1*	*Gallus gallus*	36011	6	4	201
	Ubiquitin-ribosomal protein eS31 fusion protein	*RPS27A*	*Gallus gallus*	18310	2	3	139
	Polyubiquitin-B	*UBB*	*Gallus gallus*	34348	2	3	139
	Sulfhydryl oxidase 1	*QSOX1*	*Gallus gallus*	83939	2	2	117
	Lissencephaly-1 homolog	*PAFAH1B1*	*Gallus gallus*	47204	2	2	114
	Neuroserpin	*SERPINI1*	*Gallus gallus*	46556	3	1	109
41	Transthyretin	*TTR*	*Gallus gallus*	16356	24	32	878
	Albumin	*ALB*	*Gallus gallus*	71868	7	23	327
	Acrosin	*ACR*	*Meleagris gallopavo*	38724	3	15	279
	Beta-2-microglobulin	*B2M*	*Meleagris gallopavo*	11225	5	13	268
	Fatty acid-binding protein, brain	*FABP7*	*Gallus gallus*	15031	6	11	255
	Sulfhydryl oxidase 1	*QSOX1*	*Gallus gallus*	83939	3	9	140
29	Albumin	*ALB*	*Gallus gallus*	71868	22	29	1009
	Sulfhydryl oxidase 1	*QSOX1*	*Gallus gallus*	83939	22	25	844
	Astacin-like metalloendopeptidase	*ASTL*	*Gallus gallus*	46929	17	22	575

29	Creatine kinase B-type	*CKB*	*Gallus gallus*	43129	11	18	566
	Albumin	*ALB*	*Gallus gallus*	71868	13	16	543
	Acrosin	*ACR*	*Meleagris gallopavo*	38724	12	15	434
	Transthyretin	*TTR*	*Gallus gallus*	16356	10	15	406
	Ovotransferrin	*TF*	*Gallus gallus*	79551	7	14	343
	Heat shock protein HSP 90-alpha	*HSP90AA1*	*Gallus gallus*	84406	9	13	331
	Acrosin	*ACR*	*Meleagris gallopavo*	38724	5	13	324
	L-lactate dehydrogenase B chain	*LDHB*	*Gallus gallus*	36694	8	11	323
	Clusterin	*CLU*	*Coturnix japonica*	52395	5	11	301
	Voltage-dependent anion-selective channel protein 2	*VDAC2*	*Meleagris gallopavo*	30162	5	10	288
	Annexin A2	*ANXA2*	*Gallus gallus*	38901	5	9	263
	Annexin A5	*ANXA5*	*Gallus gallus*	36290	7	8	248
	SPARC protein	*SPARC*	*Gallus gallus*	34894	4	7	192
	Golgi apparatus protein 1	*GLG1*	*Gallus gallus*	133560	4	6	189
	Glyceraldehyde-3-phosphate dehydrogenase	*GAPDH*	*Gallus gallus*	35909	3	6	177
	Transthyretin	*TTR*	*Gallus gallus*	16356	3	6	173
	Malate dehydrogenase, cytoplasmic	*MDH1*	*Gallus gallus*	36748	4	5	169
	T-complex protein 1 subunit theta	*CCT8*	*Gallus gallus*	60017	2	5	157
	14-3-3 protein zeta	*YWHAZ*	*Gallus gallus*	27929	3	4	129
	L-lactate dehydrogenase A chain	*LDHA*	*Gallus gallus*	36776	2	4	126
	1-phosphatidylinositol 4,5-bisphosphate phosphodiesterase zeta-1	*PLCZ1*	*Gallus gallus*	73285	2	3	110
	14-3-3 protein gamma	*YWHAG*	*Gallus gallus*	28384	2	3	101
	Receptor-type tyrosine-protein phosphatase gamma	*PTPRG*	*Gallus gallus*	160750	2	3	98
12	Albumin	*ALB*	*Gallus gallus*	71868	14	21	799
	Acrosin	*ACR*	*Meleagris gallopavo*	38724	11	22	608
	Creatine kinase B-type	*CKB*	*Gallus gallus*	43129	10	18	425
	Tubulin beta-4 chain	*TBB4*	*Gallus gallus*	50844	10	16	296
	Beta-2-microglobulin	*B2M*	*Meleagris gallopavo*	11225	6	13	282
	Fatty acid-binding protein, brain	*FABP7*	*Gallus gallus*	15031	5	11	280
	Cystatin	*CYT*	*Gallus gallus*	15562	8	16	268
	Ras-related protein Rab-2A	*RAB2A*	*Gallus gallus*	23678	4	12	240
	Tubulin beta-5 chain	*TBB5*	*Gallus gallus*	50395	8	12	236
	Lysozyme C	*LYZ*	*Gallus gallus*	16741	2	11	226

12	Astacin-like metalloendopeptidase	*ASTL*	*Gallus gallus*	46929	6	10	222
	Vesicle-trafficking protein SEC22b	*SEC22B*	*Gallus gallus*	24873	4	8	188
	Ovotransferrin	*TF*	*Gallus gallus*	79551	3	6	165
	Transthyretin	*TTR*	*Gallus gallus*	16356	3	5	145
	Gelsolin	*GSN*	*Gallus gallus*	86120	3	4	145
	Cytochrome c	*CYC*	*Gallus gallus*	11817	4	3	142
	Apolipoprotein A-I	*APOA1*	*Gallus gallus*	30661	5	3	130
	Ig lambda chain C region	*LAC*	*Gallus gallus*	11525	2	2	115
	Prosaposin	*PSAP*	*Gallus gallus*	59444	2	1	106
	Glyceraldehyde-3-phosphate dehydrogenase	*GAPDH*	*Gallus gallus*	35909	2	1	98

Table S3. Proteins identified by SDS-PAGE and nano LC-MS/MS in spermatozoa derived from good fertility (GF) ejaculates.

Molecular weight [kDa]	Identified protein	Gene	Species	Molecular weight (Mascot) [Da]	No. of matched peptides	Sequence coverage [%]	Score
25	Albumin	*ALB*	*Gallus gallus*	71868	21	41	1067
	Peroxiredoxin-6	*PRDX6*	*Gallus gallus*	25075	19	32	1024
	Acrosin	*ACR*	*Meleagris gallopavo*	38724	17	26	950
	Creatine kinase B-type	*CKB*	*Gallus gallus*	43129	16	24	913
	Astacin-like metalloendopeptidase	*ASTL*	*Gallus gallus*	46929	14	27	888
	Triosephosphate isomerase	*TPI1*	*Gallus gallus*	26832	17	18	807
	Phosphoglycerate mutase 1	*PGAM1*	*Gallus gallus*	29051	15	18	767
	Sulfhydryl oxidase 1	*QSOX1*	*Gallus gallus*	83939	14	19	702
	Carbonic anhydrase 2	*CA2*	*Gallus gallus*	29388	13	16	634
	Clusterin	*CLU*	*Coturnix japonica*	52395	13	19	600
	Glyceraldehyde-3-phosphate dehydrogenase	*GAPDH*	*Gallus gallus*	35909	12	14	548
	Adenylate kinase isoenzyme 1	*AK1*	*Gallus gallus*	21783	12	14	534
	Tubulin beta-1 chain	*TBB1*	*Gallus gallus*	50333	12	13	509
	Tubulin beta-2 chain	*TBB2*	*Gallus gallus*	50377	11	13	499
	Collagen alpha-1(XII) chain	*COL12A1*	*Gallus gallus*	341740	10	9	380
	Apolipoprotein A-I	*APOA1*	*Gallus gallus*	30661	9	6	305
	Glutathione S-transferase 5	*GST5*	*Gallus gallus*	25282	9	10	287
	Tubulin alpha-1 chain	*TUBA1*	*Gallus gallus*	46385	9	9	284
	14-3-3 protein zeta	*YWHAZ*	*Gallus gallus*	27929	9	8	278
	Mitochondria-eating protein	*SPATA18*	*Gallus gallus*	54997	7	9	276
	Ovotransferrin	*TF*	*Gallus gallus*	79551	8	8	269
	Actin, cytoplasmic 2	*ACTG1*	*Gallus gallus*	42108	7	11	264
	Actin, cytoplasmic type 5	*ACT5*	*Gallus gallus*	42151	7	11	264
	Ras-related protein Rab-5B	*RAB5B*	*Gallus gallus*	23828	3	10	206
	Ras-related protein Rab-5C	*RAB5C*	*Gallus gallus*	23767	3	9	198
	Ras-related protein Rab-10	*RAB10*	*Gallus gallus*	22763	2	11	158
	Cytochrome b-c1 complex subunit Rieske, mitochondrial	*UQCRFS1*	*Gallus gallus*	29710	1	4	102
	Heat shock protein HSP 90-alpha	*HSP90AA1*	*Gallus gallus*	84406	1	3	99

18	Tubulin beta-3 chain	*TBB3*	*Gallus gallus*	50285	24	40	1850
	Tubulin beta-2 chain	*TBB2*	*Gallus gallus*	50377	26	22	1608
	Tubulin beta-7 chain	*TBB7*	*Gallus gallus*	50095	26	21	1513
	Tubulin beta-4 chain	*TBB4*	*Gallus gallus*	50844	18	17	1254
	Tubulin beta-5 chain	*TBB5*	*Gallus gallus*	50395	12	16	1028
	Tubulin alpha-5 chain	*TBA5*	*Gallus gallus*	50715	13	10	877
	Tubulin alpha-4 chain	*TBA4*	*Gallus gallus*	36483	9	9	465
	ATP synthase subunit beta, mitochondrial	*ATP5B*	*Gallus gallus*	56650	10	11	368
	Creatine kinase B-type	*CKB*	*Gallus gallus*	43129	8	9	281
	Tubulin alpha-2 chain	*TBA2*	*Gallus gallus*	50715	7	9	277
	Heat shock cognate 71 kDa protein	*HSPA8*	*Gallus gallus*	71011	6	8	203
	Creatine kinase S-type, mitochondrial	*CKMT2*	*Gallus gallus*	47510	5	7	188
	Heat shock 70 kDa protein	*HSP70*	*Gallus gallus*	69936	6	5	172
	Acrosin	*ACR*	*Meleagris gallopavo*	38724	5	4	126
	Voltage-dependent anion-selective channel protein 2	*VDAC2*	*Meleagris gallopavo*	30162	5	3	115
	Outer dense fibre protein 2	*ODF2*	*Gallus gallus*	96467	4	3	106
	Ubiquitin-ribosomal protein eS31 fusion protein	*RPS27A*	*Gallus gallus*	18310	4	2	101
	Polyubiquitin-B	*UBB*	*Gallus gallus*	34348	4	2	101
	Pyruvate kinase	*PKM*	*Gallus gallus*	58434	3	1	98
	$Na^{(+)}/H^{(+)}$ exchange regulatory cofactor NHE-RF1	*NHERF1*	*Gallus gallus*	36011	2	1	95

Table S4. Proteins identified by SDS-PAGE and nano LC-MS/MS in spermatozoa derived from impaired fertility (IF) ejaculates.

Molecular weight [kDa]	Identified protein	Gene	Species	Molecular weight (Mascot) [Da]	No. of matched peptides	Sequence coverage [%]	Score
107	Albumin	*ALB*	*Gallus gallus*	71868	39	41	1389
	Cysteine protease ATG4B	*ATG4B*	*Gallus gallus*	45007	36	38	1219
	Fibronectin	*FN1*	*Gallus gallus*	276669	38	36	1203
	Aminopeptidase Ey	*ANPEP*	*Gallus gallus*	109406	28	35	1145
	Acrosin	*ACR*	*Meleagris gallopavo*	38724	27	23	1026
	Collagen alpha-1(XII) chain	*COL12A1*	*Gallus gallus*	341740	27	22	957
	Probable cation-transporting ATPase 13A4	*ATP13A4*	*Gallus gallus*	135682	27	20	857
	Ovotransferrin	*TF*	*Gallus gallus*	79551	25	16	819
	Alpha-enolase	*ENO1*	*Gallus gallus*	47617	23	21	770
	Aspartate aminotransferase, cytoplasmic	*GOT1*	*Gallus gallus*	46134	12	19	532
	Creatine kinase B-type	*CKB*	*Gallus gallus*	43129	8	12	390
	Astacin-like metalloendopeptidase	*ASTL*	*Gallus gallus*	46929	10	9	371
	Clusterin	*CLU*	*Coturnix japonica*	52395	4	9	295
	L-lactate dehydrogenase B	*LDHB*	*Gallus gallus*	36694	8	4	265
	NEL protein	*NEL*	*Gallus gallus*	96096	5	5	202
	Gelsolin	*GSN*	*Gallus gallus*	86120	5	4	136
	Heat shock cognate 71 kDa protein	*HSPA8*	*Gallus gallus*	71011	6	3	104
52	Tubulin beta-3 chain	*TBB3*	*Gallus gallus*	50285	60	21	2146
	Tubulin beta-7 chain	*TBB7*	*Gallus gallus*	50095	42	16	1394
	Tubulin beta-2 chain	*TBB2*	*Gallus gallus*	50377	40	16	1336
	Tubulin beta-5 chain	*TBB5*	*Gallus gallus*	50395	35	11	1133
	Tubulin beta-4 chain	*TBB4*	*Gallus gallus*	50844	35	13	1130
	Creatine kinase B-type	*CKB*	*Gallus gallus*	43129	16	17	836
	Creatine kinase S-type, mitochondrial	*CKMT2*	*Gallus gallus*	47510	12	16	573
	Voltage-dependent anion-selective channel protein 2	*VDAC2*	*Meleagris gallopavo*	30162	9	10	493

52	Succinate dehydrogenase [ubiquinone] iron-sulphur subunit	*SDHB*	*Gallus gallus*	33374	4	8	278
	Albumin	*ALB*	*Gallus gallus*	71868	6	6	236
	Serine/threonine-protein phosphatase PP1-beta catalytic subunit	*PPP1CB*	*Gallus gallus*	37961	3	5	181
	60 kDa heat shock protein, mitochondrial	*HSPD1*	*Gallus gallus*	61105	2	4	153
	EF-hand domain-containing family member C2	*EFHC2*	*Gallus gallus*	87461	3	3	142
	Actin, cytoplasmic 1	*ACTB*	*Gallus gallus*	42052	2	2	102
	Actin, cytoplasmic 2	*ACTG2*	*Gallus gallus*	42108	2	2	102
	Actin, cytoplasmic type 5	*ACT5*	*Gallus gallus*	42151	2	2	102
49	Creatine kinase B-type	*CKB*	*Gallus gallus*	43129	55	35	2758
	Albumin	*ALB*	*Gallus gallus*	71868	42	28	1592
	Creatine kinase S-type, mitochondrial	*CKMT2*	*Gallus gallus*	47510	29	25	1333
	Tubulin beta-3 chain	*TBB3*	*Gallus gallus*	50285	36	19	1005
	Tubulin beta-5 chain	*TBB5*	*Gallus gallus*	50395	25	21	693
	Tubulin beta-4 chain	*TBB4*	*Gallus gallus*	50844	24	17	631
	Alpha-enolase	*ENOA*	*Gallus gallus*	47617	8	9	481
	Aspartate aminotransferase, cytoplasmic	*GOT1*	*Gallus gallus*	46134	9	6	442
	Acrosin	*ACR*	*Meleagris gallopavo*	38724	5	4	328
	Voltage-dependent anion-selective channel protein 2	*VDAC2*	*Meleagris gallopavo*	30162	4	5	204
	Aspartate aminotransferase, mitochondrial	*GOT2*	*Gallus gallus*	47496	7	3	177
	Actin, cytoplasmic 1	*ACTB*	*Gallus gallus*	42052	2	2	110
	Actin, cytoplasmic 2	*ACTG2*	*Gallus gallus*	42108	2	2	110
	Actin, cytoplasmic type 5	*ACT5*	*Gallus gallus*	42151	2	2	110
41	Tubulin beta chain	*TUBB4B*	*Phasianus colchicus*	50285	21	35	1366
	Creatine kinase S-type, mitochondrial	*CKMT2*	*Phasianus colchicus*	47506	13	28	1260
	Tubulin beta 3 class III	*TUBB3*	*Coturnix japonica*	67549	17	20	957
	Medium-chain specific acyl-CoA dehydrogenase, mitochondrial	*ACADM*	*Phasianus colchicus*	46557	15	24	921
	Creatine kinase	*CKB*	*Phasianus colchicus*	43031	13	19	912
	Amidohydrolase-related domain-containing protein	*DPYS*	*Meleagris gallopavo*	55395	18	27	901
	Voltage-dependent anion-selective channel protein 2	*VDAC2*	*Meleagris gallopavo*	30162	16	32	818
	Long-chain specific acyl-CoA dehydrogenase, mitochondrial	*ACADL*	*Phasianus colchicus*	48386	15	21	757
	Acetyl-CoA acyltransferase 2	*ACAA2*	*Meleagris gallopavo*	45795	17	31	683

41	Tubulin beta-3 chain	*TBB3*	*Gallus gallus*	50285	19	28	630
	Acrosin	*ACR*	*Meleagris gallopavo*	38724	11	16	626
	Tubulin alpha-4 chain	*TBA4*	*Gallus gallus*	36483	11	15	606
	Creatine kinase S-type, mitochondrial	*CKMT2*	*Gallus gallus*	47510	8	19	484
	Tubulin beta-7 chain	*TBB7*	*Gallus gallus*	50095	11	14	315
	Tubulin beta-5 chain	*TBB5*	*Gallus gallus*	50395	8	12	243
	Complex I assembly factor ACAD9, mitochondrial	*ACAD9*	*Meleagris gallopavo*	63927	12	11	206
	Tubulin beta-4 chain	*TBB4*	*Gallus gallus*	50844	8	10	202
	Aconitate hydratase, mitochondrial	*ACO2*	*Meleagris gallopavo*	82151	5	13	152
	Ubiquitin-ribosomal protein eS31 fusion protein	*RPS27A*	*Gallus gallus*	18310	3	8	143
	Polyubiquitin-B	*UBB*	*Gallus gallus*	34348	3	8	143
	Phosphopyruvate hydratase	*ENO1*	*Phasianus colchicus*	47651	6	7	136
	Creatine kinase B-type	*CKB*	*Gallus gallus*	43129	3	7	127
	Aspartate aminotransferase, mitochondrial	*GOT2*	*Gallus gallus*	47496	3	5	119
	Ubiquitin-ribosomal protein eS31 fusion protein	*RPS27A*	*Phasianus colchicus*	18310	2	10	115
	Acyl-CoA thioesterase 1	*ACOT1*	*Meleagris gallopavo*	55422	4	5	112
	Actin, cytoplasmic 1	*ACTB*	*Gallus gallus*	42052	2	2	112
	Actin, cytoplasmic 2	*ACTG2*	*Gallus gallus*	42108	2	2	112
	Actin, cytoplasmic type 5	*ACT5*	*Gallus gallus*	42151	2	2	112
	Albumin	*ALB*	*Gallus gallus*	71868	2	1	93
29	Albumin	*ALB*	*Gallus gallus*	71868	72	51	2853
	Voltage-dependent anion-selective channel protein 2	*VDAC2*	*Phasianus colchicus*	31676	23	62	2705
	Tubulin beta chain	*TUBB4B*	*Phasianus colchicus*	50285	22	37	1941
	Tubulin beta-7 chain	*TBB7*	*Gallus gallus*	50095	20	33	1376
	Voltage-dependent anion-selective channel protein 3	*VDAC3*	*Phasianus colchicus*	42852	19	34	1281
	Voltage-dependent anion-selective channel protein 1	*VDAC1*	*Gallus gallus*	32643	15	32	1244
	Voltage-dependent anion-selective channel protein 2	*VDAC2*	*Meleagris gallopavo*	30162	21	37	1052
	Creatine kinase B-type	*CKB*	*Gallus gallus*	43129	18	22	1038
	Tubulin beta-3 chain	*TBB3*	*Gallus gallus*	50285	33	19	848
	Acrosin	*ACR*	*Meleagris gallopavo*	38724	22	18	722
	Tubulin beta-4 chain	*TBB4*	*Gallus gallus*	50844	22	16	523
	Tubulin beta-5 chain	*TBB5*	*Gallus gallus*	50395	18	13	503
	Creatine kinase S-type, mitochondrial	*CKMT2*	*Gallus gallus*	47510	7	10	412
	Ig lambda chain C region	*LAC*	*Gallus gallus*	11525	8	9	330
	Apolipoprotein A-I	*APOA1*	*Gallus gallus*	30661	10	8	301

	Tubulin alpha-4 chain	*TBA4*	*Gallus gallus*	36483	6	14	301
	Phosphoinositide phospholipase C	*PLCZ1*	*Coturnix japonica*	73293	8	8	260
	Actin gamma 1	*ACTG1*	*Phasianus colchicus*	42151	13	20	206
	Phosphoinositide phospholipase C	*PLCZ1*	*Phasianus colchicus*	72414	9	9	186
	Phosphoglycerate kinase	*PGK*	*Gallus gallus*	45087	5	7	180
	ATP synthase subunit beta, mitochondrial	*ATP5B*	*Gallus gallus*	56650	6	6	171
	Outer dense fibre protein 2	*ODF2*	*Meleagris gallopavo*	103032	9	5	168
	Phosphoglycerate mutase 1	*PGAM1*	*Gallus gallus*	29051	3	5	154
	Succinate dehydrogenase [ubiquinone] iron-sulphur subunit	*SDHB*	*Gallus gallus*	33374	4	5	146
	Heat shock protein 90 alpha family class A member 1	*HSP90AA1*	*Gallus gallus*	84466	4	3	146
	Astacin-like metalloendopeptidase	*ASTL*	*Gallus gallus*	46929	3	4	132
	Tubulin alpha-2 chain	*TBA2*	*Gallus gallus*	50715	4	3	127
	Mitochondria-eating protein	*SPATA18*	*Meleagris gallopavo*	51247	4	5	119
	Glyceraldehyde-3-phosphate dehydrogenase	*GAPDH*	*Gallus gallus*	35909	3	2	111
	Sperm associated antigen 16	*SPAG16*	*Meleagris gallopavo*	29646	2	4	107
	EF-hand domain-containing family member C2	*EFHC2*	*Gallus gallus*	87461	2	1	105
	Mitochondria-eating protein	*SPATA18*	*Gallus gallus*	54997	1	1	99
	Sperm associated antigen 6	*SPAG6*	*Phasianus colchicus*	68067	2	1	94
16	Voltage-dependent anion-selective channel protein 2	*VDAC2*	*Meleagris gallopavo*	30162	26	35	1473
	Tubulin beta-7 chain	*TBB7*	*Gallus gallus*	50095	50	33	1350
	Tubulin beta-5 chain	*TBB5*	*Gallus gallus*	50395	26	29	1216
	Tubulin beta-4 chain	*TBB4*	*Gallus gallus*	50844	39	19	1102
	Creatine kinase B-type	*CKB*	*Gallus gallus*	43129	16	15	758
	Tubulin alpha-4 chain	*TBA4*	*Gallus gallus*	36483	15	13	613
	Acrosin	*ACR*	*Meleagris gallopavo*	38724	10	12	566
	ATP synthase subunit beta, mitochondrial	*ATP5B*	*Gallus gallus*	56650	11	10	478
	Creatine kinase S-type, mitochondrial	*CKMT2*	*Gallus gallus*	47510	10	11	459
	Glyceraldehyde-3-phosphate dehydrogenase	*GAPDH*	*Gallus gallus*	35909	4	10	254
	Calmodulin	*CALM*	*Gallus gallus*	16827	4	8	224
	EF-hand domain-containing family member C2	*EFHC2*	*Gallus gallus*	87461	4	8	220
	Fatty acid-binding protein, brain	*FABP7*	*Gallus gallus*	15031	4	6	199
	Succinate dehydrogenase [ubiquinone] iron-sulphur subunit	*SDHB*	*Gallus gallus*	33374	2	7	188
	Outer dense fibre protein 2	*ODF2*	*Gallus gallus*	96467	4	5	168

16	Mitochondria-eating protein	*SPATA18*	*Gallus gallus*	54997	2	5	127
	Ubiquitin-ribosomal protein eS31 fusion protein	*RPS27A*	*Gallus gallus*	18310	2	4	124
	Polyubiquitin-B	*UBB*	*Gallus gallus*	34348	2	3	124
	Cilia- and flagella-associated protein 20	*CFAP20*	*Gallus gallus*	22891	3	3	122
	Actin, cytoplasmic 1	*ACTB*	*Gallus gallus*	42052	2	2	102
	Actin, cytoplasmic 2	*ACTG2*	*Gallus gallus*	42108	2	2	102
	Actin, cytoplasmic type 5	*ACT5*	*Gallus gallus*	42151	2	2	102

Table S5. Proteins identified in seminal plasma from good fertility (GF) and impaired fertility (IF) ejaculates and involved in selected biological processes.

**Biological process (GO)**	**GF**	**IF**
Biological process involved in interspecies interaction between organisms	TF	TF
Biological regulation	CFAP20, GSN, HSP90AA1, PLCZ1, TF, YWHAB, YWHAG, YWHAE, YWHAQ, YWHAZ	CYC, FGB, GSN, HSP90AA1, NDK, PLCZ1, PSAP, SERPINI1, TF, TGFB2, YWHAG, YWHAZ
Cellular processes	ACTG1, ALB, CCT8, CFAP20, CKD, CKB, COL12A1, GAPDH, GSN, HAGH, HPRT1, HSP90AA1, PPIB, PLCZ1, QSOX1, RAB10, RAB5B, RAB5C, SDHB, TBB3, TBB5, TF, TPI1, TTR, TUBA1, YWHAB, YWHAG, YWHAQ, YWHAZ, ZPBP1	ACO1, ACTN4, ALB, ANPEP, CCT8, CKB, COL12A1, CYC, EEF2, ENO1, ENO2, FGB, GAPDH, GSN, GOT1, HSP90AA1, HSP90B1, LDHA, MDH1, NHERF1, PLCZ1, PSAP, QSOX1, TF, TGFB2, TBB5, TTR, TUBA1, YWHAG, YWHAZ
Developmental process	ACTB, ACTG1, COL12A1, GSN, SPARC, ZPBP1	ACTN4, COL12A1, GSN, PAFAH1B1, PSAP, SPARC
Growth	-	PSAP
Homeostatic process	APOA1, FTH, PRDX6	APOA1
Immune system process	TF	TF
Localisation	ALB, APOA1, FTH, RAB10, RAB5B, RAB5C, RBP4, TF, YWHAB, YWHAG, YWHAE, YWHAQ, YWHAZ	ALB, APOA1, FABP7, LAMP1, NHERF1, PAFAH1B1, RAB2A, TF, YWHAG, YWHAZ
Metabolic process	APOA1, CCKB, CCT8, CKMT2, GAPDH, HAGH, HPRT1, HSP90AA1, PPIB, PSMB5, QSOX1, RPS27A, SDHB, SPATA18, TPI1, TTR, UBB	ACO1, ANPEP, APOA1, BLMH, CCT8, CKB, CYC, EEF1A, EEF2, EIF4A2, ENO1, ENO2, FGB, GAPDH, GOT1, HSP90AA1, HSP90B1, LDHA, LDHB, MDH1, PPIB, QSOX1, RPS27A, TTR, UBB
Multicellular organismal process	ACTB, ACTG1, COL12A1, GSN, PLCZ1, ZPBP1	COL12A1, FGB, GSN, PAFAH1B1, PLCZ1, PSAP
Reproduction	PLCZ1, ZPBP1	PAFAH1B1, PLCZ1, PSAP
Reproductive process	PLCZ1, ZPBP1	PAFAH1B1, PLCZ1, PSAP
Response to stimulus	HSP90AA1, TF, YWHAB, YWHAG, YWHAE, YWHAQ, YWHAZ	BLMH, FGB, HSP90AA1, HSP90B1, PSAP, TF, TGFB2, YWHAG, YWHAZ

Table S6. Proteins identified in spermatozoa from good fertility (GF) and impaired fertility (IF) ejaculates and involved in selected biological processes.

**Biological process (GO)**	**GF**	**IF**
Biological process involved in interspecies interaction between organisms	TF	TF
Biological regulation	HSP90AA1, ODF2, TF, YWHAZ	CFAP20, GSN, HSPD1, ODF2, TF
Cellular processes	ACTG1, ALB, CKB, CKMT2, COL12A1, GAPDH, GST5, HSP90AA1, NHERF1, PKM, QSOX1, RAB10, RAB5B, RAB5C, SPATA18, TF, TBB1, TBB3, TBB5, TPI1, TUBA1, YWHAZ	ACTB, ALB, ANPEP, ATG4B, ATP13A4, CALM, CFAP20, CKB, CKMT2, COL12A1, ENO1, FN1, GAPDH, GOT1, GOT2, GSN, HSPD1, LDHB, SDHB, SPATA18, TBB3, TBB5, TF
Developmental process	ACTG1, COL12A1	ACTB, COL12A1, FN1, GSN
Homeostatic process	APOA1, PRDX6	APOA1, ATP13A4
Immune system process	TF	HSPD1, TF, SPAG6, SPAG16
Localisation	ALB, APOA1, NHERF1, RAB10, RAB5B, RAB5C, TF, YWHAZ	ALB, APOA1, ATP13A4, CALM, FABP7, HSPD1, TF
Metabolic process	APOA1, CKB, CKMT2, GAPDH, GST5, HSP90AA1, PKM, QSOX1, RPS27A, SPATA18, TPI1, UBB	ANPEP, APOA1, ATG4B, CKB, CKMT2, ENO1, GAPDH, GOT1, GOT2, HSPD1, LDHB, RPS27A, SDHB, SPATA18, UBB
Multicellular organismal process	ACTG1, COL12A1	ACTB, COL12A1, FN1, GSN, HSPD1
Response to stimulus	HSP90AA1, PKM, TF, YWHAZ	HSPD1, TF
